# Opportunities to reduce antibiotic prescribing for patients with COPD in primary care: a cohort study using electronic health records from the Clinical Practice Research Datalink (CPRD)

**DOI:** 10.1093/jac/dkz411

**Published:** 2019-10-09

**Authors:** Patrick Rockenschaub, Arnoupe Jhass, Nick Freemantle, Anna Aryee, Meena Rafiq, Andrew Hayward, Laura Shallcross

**Affiliations:** 1 Institute of Health Informatics, University College London, 222 Euston Rd, London NW1 2DA, UK; 2 Primary Care & Population Health, University College London, Rowland Hill Street, London NW3 2PF, UK; 3 Institute of Clinical Trials and Methodology, University College London, 90 High Holborn, London WC1V 6LJ, UK; 4 Institute of Epidemiology & Healthcare, University College London, 1-19 Torrington Place, London WC1E 7HB, UK

## Abstract

**Background:**

In primary care there is uncertainty about which patients with acute exacerbations of COPD (AECOPD) benefit from antibiotics.

**Objectives:**

To identify which types of COPD patients get the most antibiotics in primary care to support targeted antibiotic stewardship.

**Methods:**

Observational study of COPD patients using a large English primary care database with 12 month follow-up. We estimated the incidence of and risk factors for antibiotic prescribing relative to the number of AECOPD during follow-up, considering COPD severity, smoking, obesity and comorbidity.

**Results:**

From 157 practices, 19594 patients were diagnosed with COPD, representing 2.6% of patients and 11.5% of all prescribed antibiotics. Eight hundred and thirty-three (4.5%) patients with severe COPD and frequent AECOPD were prescribed six to nine prescriptions per year and accounted for 13.0% of antibiotics. Individuals with mild to moderate COPD and zero or one AECOPD received one to three prescriptions per year but accounted for 42.5% of all prescriptions. In addition to COPD severity, asthma, chronic heart disease, diabetes, heart failure and influenza vaccination were independently associated with increased antibiotic use.

**Conclusions:**

Patients with severe COPD have the highest rates of antibiotic prescribing but most antibiotics are prescribed for patients with mild to moderate COPD. Antibiotic stewardship should focus on the dual goals of safely reducing the volume of prescribing in patients with mild to moderate COPD, and optimizing prescribing in patients with severe disease who are at significant risk of drug resistance.

## Introduction

Reducing inappropriate antibiotic prescribing is a global priority to curb the emergence of antimicrobial resistance (AMR). In England, more than three-quarters of human antibiotics are prescribed by the patient’s GP,^1^ where stewardship measures have focused on reducing prescribing for minor infections in the general population. However, the highest rates of GP antibiotic prescribing are seen in patients with chronic diseases, and few studies have investigated whether there is scope to reduce antibiotic prescribing in these patients.^2^

Patients with COPD receive three times more antibiotic prescriptions in primary care than the general population, mainly for the treatment of acute exacerbations (AECOPD).^2^ AECOPD occur approximately 0.5–3.5 times per year and are characterized by a rapid deterioration in breathlessness, cough and increased production and purulence of sputum.^3^ Around half of all acute exacerbations are thought to be bacterially mediated, with the remainder caused by viral infections or environmental triggers.^4^ Although this implies that only half of AECOPD require antibiotic treatment, it is difficult for GPs to differentiate between patients who will or will not benefit from antibiotic treatment owing to factors such as diagnostic uncertainty, patient expectation and the setting in which the consultation takes place.^5^ Antibiotic treatment may shorten the duration of symptoms and/or reduce the risk of hospital admission, but this has to be weighed against the risk of resistance following repeated courses of potentially unnecessary antibiotics.^4^

Recent guidelines have highlighted uncertainty around which groups of people with AECOPD may benefit most from antibiotics, emphasizing the need to consider the severity of symptoms and risk of adverse outcomes when deciding whether to prescribe.^6^ However, there is little evidence on how to weight these different factors when making the decision to prescribe. To inform appropriate stewardship interventions, we used electronic health records (EHRs) from primary care to identify which COPD patients receive most antibiotics.

## Methods

### Data source

We analysed individual-level EHRs from the Clinical Practice Research Datalink (CPRD), a large, pseudonymized, retrospective database of primary care records from the UK.^7^ CPRD includes data for 4.4 million actively registered patients (roughly 7% of the UK population) and is broadly representative of the general population. Data encompass symptoms, prescriptions, diagnoses, referrals to specialist care, and diagnostic tests. All clinical information is recorded via Read codes, a hierarchical medical coding system.

A subset of CPRD practices (75% of English practices, 58% of UK practices) are linked to data on NHS hospital admissions and census data.^7^ This analysis includes data from all English practices in CPRD that were linked to hospital and census data. Ethics approval was obtained from CPRD’s Independent Scientific Advisory Committee (ISAC-Nr.: 17_048).

### Study population

The study population included patients aged between 35 and 110 years provided they were eligible for record linkage, registered for the entire year 2015, had at least 12 months registration before entering the study, and had valid records for gender and socio-economic status [Index of Multiple Deprivation (IMD) 2015]; Figure S1, available as Supplementary data at *JAC* Online.^7^ Individuals were excluded if they lacked a record of being a current or ex-smoker.^8^ COPD diagnosis was defined as the first consultation with a relevant Read code based on the Quality and Outcomes Framework (Table S1). Patients entered the study on 1 January 2015 and exited on 31 December 2015. The denominator was the total number of person-years contributed by patients in the sample, excluding time periods when the patient was hospitalized.

### Measurement of exposures and covariates

COPD severity was classified by three methods, using the latest measurement in the year preceding study entry. Forced expiratory volume in 1 s as a percentage of that predicted (FEV1) was used as an objective measure of airflow limitation (Tables S2 and S3), grouping patients into Global Initiative for Chronic Obstructive Lung Disease (GOLD) categories GOLD 1 (FEV1 ≥80%), GOLD 2 (50%–80%), GOLD 3 (30%–<50%) and GOLD 4 (<30%).^9^ The self-reported MRC dyspnoea scale was used to measure the impact of the patient’s breathlessness (Table S4), ranging from MRC 1 (breathless only with strenuous exercise) to MRC 5 (too breathless to leave the house).^10^ Finally, we estimated the number of acute exacerbations in the year preceding the study based on a combination of prescribing (antibiotics and oral corticosteroids), symptoms and diagnosis codes validated in a previous publication (Table S5).^11^ Patients were grouped into those with no exacerbation, one, two, or three or more exacerbations managed in primary care, and one or more exacerbations associated with hospital admission.^12^ The same approach was applied to estimate the number of AECOPD during follow-up.

We used Read codes to identify patients with selected comorbidities, smoking and obesity since these factors are relevant to the decision to prescribe an antibiotic (Table S1). Patients were classified as obese if the latest record of BMI in the previous 5 years was >30 kg/m^2^. Patients were considered current smokers if their latest recorded smoking status within 5 years suggested continued smoking, and as ex-smokers if the latest record indicated they had stopped smoking. Patients labelled non-smokers were re-classified as ex-smokers if they had any preceding record of smoking.

### Patterns of antibiotic prescribing and clinical indication

We measured prescriptions of systemic antibiotics included in Chapter 5.1 of the BNF, excluding anti-tuberculosis (5.1.9) and anti-leprotic drugs (5.1.10). We investigated how the reason for the prescription and the duration of prescription varied according to the severity of COPD. Since the reason for the prescription is not well recorded in EHRs, we used prescribing data combined with national guidance to categorize prescriptions into first-line (amoxicillin, doxycycline, clarithromycin), second-line (co-amoxiclav, co-trimoxazole, levofloxacin) or prophylactic (azithromycin) treatment for AECOPD.^13^ We also investigated the frequency of prescribing for common non-respiratory infections (urinary and skin and soft tissue), using prescriptions for nitrofurantoin and flucloxacillin as proxies for each of these indications respectively.

We categorized the duration of prescribing, excluding prescriptions that could be explicitly linked to a non-respiratory indication to avoid misclassifying prophylaxis for other infections as long-term prescribing for COPD. Diagnostic codes entered on the same day as the antibiotic prescription were treated as potential indications and used to determine whether the prescription represented an acute first course (no prescription for the same indication in the prior 30 days), an acute second course (at least one prescription for the same indication in the prior 30 days), or a continuous prescribing sequence of <6 or ≥6 months, based on an existing codelist (Table S6).^14^ Continuous prescriptions were those either not fulfilling the criteria for acute courses or were explicitly labelled as part of a prescribing sequence (see Supplementary data for a more detailed description).

### Statistical analysis

We calculated crude and adjusted rates of antibiotic prescribing by demography, COPD severity, comorbidity, smoking status and obesity using a negative binomial regression model with random intercept terms for general practices. To investigate prescribing patterns, we estimated the proportion and rates of prescribing for each type of antibiotic and clinical indication, stratified by the number of AECOPD during follow-up. To investigate whether COPD severity at study entry predicts antibiotic prescribing during follow-up, we calculated the antibiotic prescribing rate comparing severity based on FEV1, MRC dyspnoea scale, and the number of AECOPD in the year preceding study entry (baseline). We generated mosaic plots to visualize the relationship between total antibiotic prescribing, COPD severity and the rate of antibiotic prescribing per patient, displaying rates of prescribing for an ‘average’ COPD patient (male, age 60–70 years, IMD 3). A non-COPD reference population was obtained by matching up to four non-COPD patients per case based on year of birth, sex and general practice.

Crude analyses excluded missing data (complete case analysis). For the adjusted regression models, multiple imputation running 20 chains with 40 iterations was used to impute missing variables for FEV1 and MRC dyspnoea scale (Tables S7–S11 and Figures S2–S7). The analysis was replicated using data from 2013 and 2014. All analyses were performed using the statistical software R version 3.4.3 for Windows.^15^ Regression modelling was done using the R package *glmmTMB* (version 0.2.2) and multiple imputation was performed using the R package *mice* (version 3.3.0).

## Results

Across 157 practices, 759425 individuals were eligible for inclusion in the study and 19594 (2.6%) met diagnostic criteria for COPD. The mean age of COPD patients was 71 years and 46.4% of patients were female (Table 1). Approximately 70%–80% of patients had mild to moderate COPD (GOLD 1–2 or MRC 1–3). FEV1 or MRC had not been measured in the year preceding study entry in 39% and 26% of patients respectively, but had been recorded in the prior 2 years for 92% of patients. There were no records of AECOPD for 12457 (63.6%) patients during the 12 months before study entry, 5761 (29.4%) had at least one exacerbation managed in primary care and 1376 (7.0%) had an exacerbation requiring hospital admission. The commonest comorbidities were asthma (32.0%) and obesity (28.5%) and 39.1% of patients were current smokers (Table 1).

**Table 1.** Multivariate analysis of the association between the baseline characteristics of the COPD cohort and rate of antibiotic prescribing in the following 12 months

		Prescriptions			
Patient characteristics	Patients, *n* (%)	*n* (%)	crude rate (95% CI)	unadjusted RR (95% CI)	adjusted RR^a^ (95% CI)
Total					
Age, years	19594 (100.0)	57939 (100.0)	2.88 (2.77–3.00)		
35–<50	624 (3.2)	1469 (2.5)	2.33 (2.08–2.62)	0.80 (0.71–0.90)	0.81 (0.69–0.96)
50–<60	2409 (12.3)	6351 (11.0)	2.53 (2.37–2.71)	0.87 (0.81–0.93)	0.88 (0.80–0.96)
60–<70^b^	5726 (29.2)	17247 (29.8)	2.92 (2.77–3.07)	1	1
70–80	6692 (34.2)	20383 (35.2)	2.97 (2.83–3.12)	1.02 (0.97–1.07)	1.01 (0.94–1.07)
>80	4143 (21.1)	12489 (21.6)	2.97 (2.81–3.14)	1.02 (0.96–1.08)	0.94 (0.87–1.01)
Female	9088 (46.4)	29772 (51.4)	3.21 (3.09–3.34)	1.23 (1.19–1.28)	1.29 (1.20–1.38)
IMD					
1 (least deprived)	3078 (15.7)	8492 (14.7)	2.68 (2.50–2.88)	0.93 (0.87–1.00)	0.98 (0.92–1.05)
2	3432 (17.5)	9718 (16.8)	2.71 (2.54–2.89)	0.94 (0.88–1.01)	0.97 (0.91–1.04)
3^b^	4016 (20.5)	11987 (20.7)	2.87 (2.71–3.04)	1	1
4	4582 (23.4)	13749 (23.7)	3.03 (2.85–3.22)	1.05 (0.99–1.12)	1.04 (0.98–1.11)
5 (most deprived)	4486 (22.9)	13993 (24.2)	3.05 (2.86–3.25)	1.06 (0.99–1.13)	1.03 (0.96–1.09)
FEV1, GOLD criteria					
1	1854 (15.4)	4119 (12.0)	2.16 (2.02–2.32)	0.86 (0.81–0.93)	
2^b^	6771 (56.3)	17314 (50.6)	2.50 (2.38–2.63)	1	
3	2837 (23.6)	10079 (29.4)	3.46 (3.27–3.67)	1.38 (1.31–1.46)	
4	554 (4.6)	2726 (8.0)	4.86 (4.36–5.43)	1.94 (1.74–2.17)	
missing	7578				
MRC dyspnoea scale					
1	2349 (16.1)	3917 (9.0)	1.64 (1.53–1.75)	0.70 (0.65–0.75)	0.73 (0.68–0.78)
2^b^	5815 (40.0)	14043 (32.2)	2.35 (2.23–2.46)	1	1
3	3910 (26.9)	13504 (31.0)	3.40 (3.23–3.58)	1.45 (1.37–1.53)	1.44 (1.37–1.52)
4	2095 (14.4)	9792 (22.5)	4.67 (4.38–4.98)	1.99 (1.87–2.12)	1.95 (1.83–2.09)
5	381 (2.6)	2296 (5.3)	6.12 (5.38–6.95)	2.61 (2.29–2.96)	2.49 (2.19–2.82)
missing	5044				
Frequency of AECOPD at baseline					
0^b^	12457 (63.6)	25677 (44.3)	2.00 (1.92–2.09)	1	
1 in primary care	3374 (17.2)	10576 (18.3)	3.12 (2.97–3.28)	1.56 (1.48–1.64)	
2 in primary care	1270 (6.5)	5558 (9.6)	4.41 (4.10–4.74)	2.20 (2.05–2.36)	
≥3 in primary care	1117 (5.7)	7340 (12.7)	6.68 (6.20–7.20)	3.33 (3.09–3.59)	
≥1 requiring hospital admission	1376 (7.0)	8788 (15.2)	6.44 (6.02–6.89)	3.21 (3.00–3.44)	
Asthma	6268 (32.0)	21894 (37.8)	3.42 (3.29–3.57)	1.30 (1.25–1.36)	1.22 (1.17–1.27)
Chronic heart disease	3071 (15.7)	10479 (18.1)	3.32 (3.15–3.50)	1.18 (1.12–1.25)	1.08 (1.02–1.14)
Chronic kidney disease	3052 (15.6)	9936 (17.1)	3.25 (3.08–3.43)	1.15 (1.09–1.22)	1.01 (0.96–1.07)
Diabetes	3066 (15.6)	10414 (18.0)	3.31 (3.14–3.49)	1.18 (1.12–1.24)	1.07 (1.02–1.13)
Heart failure	1095 (5.6)	4295 (7.4)	3.88 (3.57–4.21)	1.37 (1.26–1.49)	1.17 (1.08–1.27)
PAD^c^	1184 (6.0)	3768 (6.5)	3.13 (2.89–3.39)	1.09 (1.01–1.18)	1.06 (0.97–1.14)
Stroke	1650 (8.4)	5497 (9.5)	3.26 (3.05–3.49)	1.14 (1.07–1.23)	1.06 (0.99–1.13)
Obesity	5587 (28.5)	18267 (31.5)	3.17 (3.04–3.30)	1.14 (1.10–1.19)	1.01 (0.97–1.05)
Smoking	7670 (39.1)	21047 (36.3)	2.65 (2.54–2.75)	0.87 (0.84–0.91)	0.91 (0.88–0.95)
Flu vaccination	15736 (80.3)	48546 (83.8)	3.01 (2.87–3.16)	1.27 (1.21–1.34)	1.23 (1.17–1.29)

a Adjusted for all other baseline variables and random effects on the practice level. Not adjusted for FEV1 and AECOPD. Missing variables of MRC imputed using multiple imputation.

b Reference category.

c Peripheral arterial disease.

Patients with COPD were prescribed a total of 57939 antibiotic courses in 2015, accounting for 11.5% (57939/504902) of the total amount of antibiotic prescriptions that were issued by GPs in our dataset. The mean antibiotic prescription rate per COPD patient per year was 2.88 (95% CI 2.77–3.00). In the adjusted analysis, increasing age (>60 years), female gender [rate ratio (RR) 1.29; 95% CI 1.20–1.38] and comorbidities including asthma (RR 1.22; 95% CI 1.17–1.27), coronary heart disease (RR 1.08; 95% CI 1.02–1.14), diabetes (RR 1.07; 95% CI 1.02–1.13) and heart failure (RR 1.17; 95% CI 1.08–1.27) were associated with higher rates of antibiotic prescribing (Tables 1 and S12). Rates were also higher in patients who had been vaccinated against influenza (RR 1.23; 95% CI 1.17–1.29) but current smokers received 9% fewer antibiotics on average (RR 0.91; 95% CI 0.88–0.95).

### Patterns of antibiotic prescribing to patients with COPD

**Figure 1.**
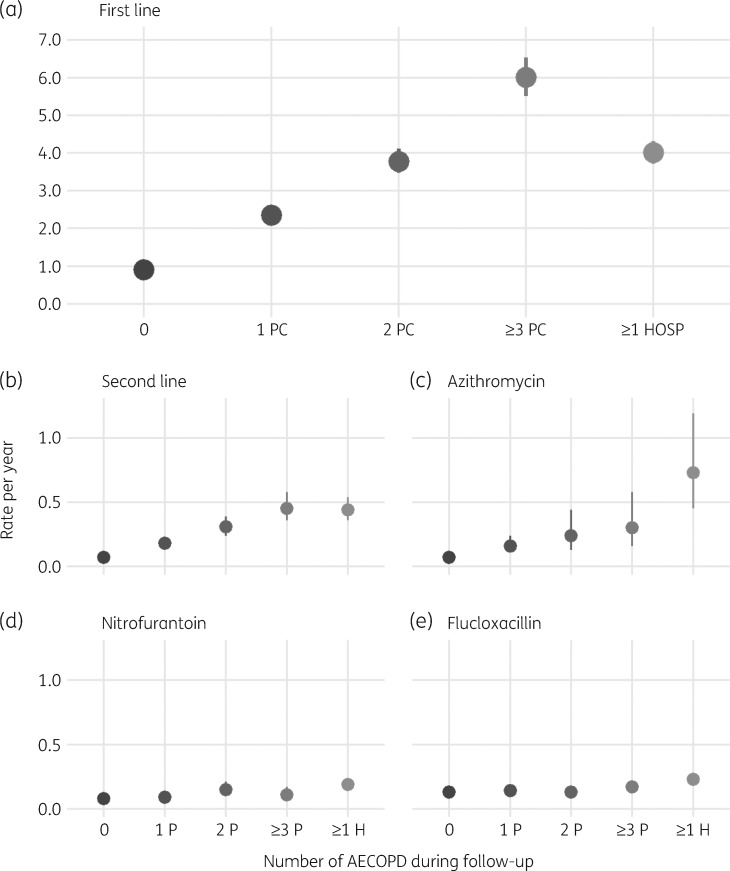
Average annual rate of first-line, second-line and specific antibiotic therapies by the number of AECOPD during follow-up (filled circles). (a) First-line antibiotic therapy. (b) Second-line antibiotic therapy. (c) Azithromycin as a marker of prophylactic antibiotic use in COPD. (d) Nitrofurantoin as a marker of prescribing for urinary tract infection. (e) Flucloxacillin as a marker of prescribing for skin and soft tissue infections. PC or P, AECOPD managed in primary care; HOSP or H, AECOPD requiring hospitalization.

The rate of prescribing of first- and second-line therapy for COPD increased with the number of AECOPD during follow-up (Figure 1). Patients who had been hospitalized had lower rates of prescribing than those with three or more AECOPD managed in primary care but had the highest usage of azithromycin. Prescribing rates for nitrofurantoin and flucloxacillin were comparable in patients with and without AECOPD.

**Figure 2.**
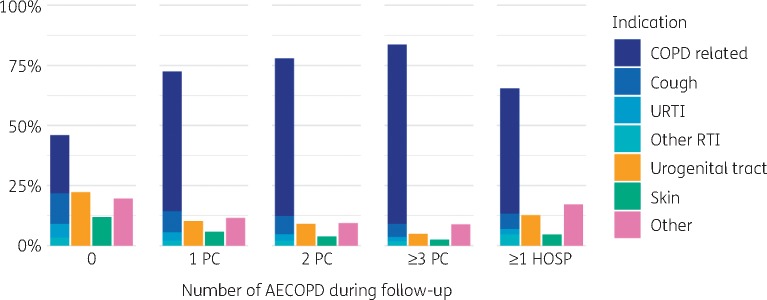
Indication for the antibiotic prescription, grouped by the number of AECOPD during follow-up. PC, AECOPD managed in primary care; HOSP, AECOPD requiring hospitalization. This figure appears in colour in the online version of *JAC* and in black and white in the print version of *JAC*.

For 37.3% (21619) of prescriptions there was no link to an indication. The indication was more likely to be missing in patients with exacerbations associated with hospital admission (4475; 41.0%) or in individuals with no record of AECOPD during follow-up (10690; 47.0%). Respiratory conditions accounted for 23630 (65.1%) of prescriptions with a recorded indication. Urogenital tract (4962; 13.7%) and skin and soft tissue infections (3355; 9.2%) were the most common non-respiratory reasons for prescribing (Figure 2).

Limiting the analysis to prescriptions for a potential respiratory indication (i.e. explicitly labelled respiratory or no indication), <3.7% of patients with at least one AECOPD managed in primary care during follow-up were not treated with antibiotics compared with more than half (56.7%) of patients without a record of AECOPD (Table 2). Initial prescribing accounted for 47.9% of prescriptions for an acute episode, i.e. the first time the patient had received an antibiotic for that episode of infection. Only 2.8% of patients were on continuous antibiotic treatment for ≥6 months, but this accounted for 11.2% of prescriptions for a respiratory indication. Long-term continuous prescribing was most prevalent in patients who had been hospitalized for AECOPD, accounting for 15.9% of all their prescriptions.

**Table 2.** Patterns of short- and long-term antibiotic use with a respiratory indication, stratified by the number of AECOPD during follow-up comparing patients and prescriptions

	Number of AECOPD during follow-up					
		Primary care				
	0	1	2	≥3	hospital ≥1	all
Number/percentage of patients						
total	12701 (100)	3049 (100)	1137 (100)	991 (100)	1716 (100)	19594 (100)
with no prescribing	7202 (56.7)	112 (3.7)	6 (0.5)	^a^ (<0.5)	228 (13.3)	7549 (38.5)
with prescribing <6 months	5242 (41.3)	2850 (93.5)	1088 (95.7)	948 (95.7)	1363 (79.4)	11490 (58.6)
with prescribing ≥6 months	257 (2.0)	87 (2.9)	43 (3.8)	43 (4.3)	125 (7.3)	555 (2.8)
Number/percentage of prescribing						
total	16238 (100)	8755 (100)	5026 (100)	6535 (100)	8695 (100)	45249 (100)
acute first course	7529 (46.4)	4961 (56.7)	2783 (55.4)	2971 (45.5)	3420 (39.3)	21664 (47.9)
acute second course	638 (3.9)	499 (5.7)	491 (9.8)	650 (9.9)	520 (6.0)	2798 (6.2)
continuous (<6 months)	5908 (36.4)	2476 (28.3)	1378 (27.4)	2593 (39.7)	3376 (38.8)	15731 (34.8)
continuous (≥6 months)	2163 (13.3)	819 (9.4)	374 (7.4)	321 (4.9)	1379 (15.9)	5056 (11.2)

a Values under 5 were suppressed and patients were added to the largest category to prevent re-identification.

### Antibiotic prescribing and COPD severity

All three measures of COPD severity were associated with increased rates of antibiotic prescribing (Table 3). For ‘average’ patients (male, 60–70 years, IMD 3) without a recorded exacerbation during follow-up, average prescribing increased from 1.20 (95% CI 1.11–1.31) antibiotics per year in GOLD 1 to 2.95 (95% CI 2.61–3.34) in GOLD 4. Using the MRC dyspnoea scale, the antibiotic prescribing rate increased from 0.97 (95% CI 0.90–1.05) in MRC 1 to 3.39 (95% CI 2.87–4.01) in MRC 5, and from 1.31 (95% CI 1.24–1.38) in patients with no exacerbation during baseline to 3.37 (95% CI 2.92–3.89) in patients with three or more moderate exacerbations managed in primary care. Across all measures of COPD severity, rates of antibiotic prescribing in patients with three or more exacerbations in primary care during follow-up ranged from 6.0–6.4 prescriptions per year in patients with GOLD 1 or MRC 1 to 9.1–9.2 prescriptions per year among patients with GOLD 4 or MRC 5. Patients with more than three AECOPD during baseline and a hospitalization during follow-up had the highest estimated rate of prescribing, with 10.9 (95% CI 8.8–13.5) antibiotic prescriptions per year. Results for the complete case analysis and the years 2013 and 2014 were similar (Table S13–S15). In comparison, the rate of prescribing in the age-, sex- and practice-matched group without COPD (*N*=76440) was 0.60 (95% CI 0.57–0.62) prescriptions per year.

**Table 3.** Rate of antibiotic prescribing (95% CI) according to the number of AECOPD during follow-up, stratifying by FEV1, MRC scale and number of AECOPD at baseline

	Number of AECOPD during follow-up				
		Primary care			
	0	1	2	≥3	hospital ≥1
FEV1, GOLD criteria					
1	1.20 (1.11–1.31)	2.64 (2.33–2.98)	4.74 (3.95–5.69)	6.42 (5.20–7.91)	5.30 (4.21–6.66)
2	1.40 (1.33–1.49)	3.06 (2.83–3.30)	4.57 (4.12–5.06)	6.89 (6.19–7.67)	5.46 (4.93–6.04)
3	1.92 (1.79–2.07)	3.59 (3.23–3.99)	5.40 (4.68–6.23)	7.80 (6.72–9.05)	6.11 (5.46–6.83)
4	2.95 (2.61–3.34)	5.04 (4.00–6.35)	5.50 (4.05–7.47)	9.09 (6.94–11.92)	7.35 (6.22–8.68)
MRC dyspnoea scale					
1	0.97 (0.90–1.05)	2.50 (2.20–2.83)	3.99 (3.23–4.93)	6.37 (4.96–8.18)	4.06 (3.16–5.22)
2	1.33 (1.26–1.42)	2.90 (2.67–3.14)	4.21 (3.74–4.74)	6.66 (5.84–7.60)	4.67 (4.13–5.28)
3	1.86 (1.73–1.98)	3.47 (3.15–3.81)	5.36 (4.72–6.09)	7.40 (6.52–8.40)	6.19 (5.57–6.89)
4	2.61 (2.41–2.83)	4.47 (3.95–5.05)	6.47 (5.46–7.66)	9.15 (7.81–10.71)	7.02 (6.28–7.86)
5	3.39 (2.87–4.01)	5.26 (3.84–7.21)	6.97 (4.71–10.30)	9.21 (6.28–13.52)	9.07 (7.41–11.09)
Number of AECOPD during baseline period					
0	1.31 (1.24–1.38)	2.86 (2.66–3.07)	4.45 (3.98–4.97)	6.02 (5.17–7.01)	4.15 (3.77–4.57)
1 in primary care	1.85 (1.72–1.99)	3.54 (3.22–3.89)	4.47 (3.89–5.15)	6.51 (5.56–7.63)	5.25 (4.58–6.02)
2 in primary care	2.85 (2.57–3.18)	3.81 (3.32–4.37)	5.02 (4.18–6.02)	7.43 (6.25–8.84)	6.39 (5.24–7.80)
≥3 in primary care	3.37 (2.92–3.89)	4.83 (4.11–5.69)	6.67 (5.64–7.90)	8.62 (7.61–9.76)	9.60 (8.09–11.40)
≥1 in hospital	3.31 (2.97–3.68)	4.64 (3.91–5.50)	7.27 (5.87–9.00)	10.86 (8.76–13.47)	8.36 (7.53–9.28)

### Identifying potential target groups for antibiotic stewardship

**Figure 3.**
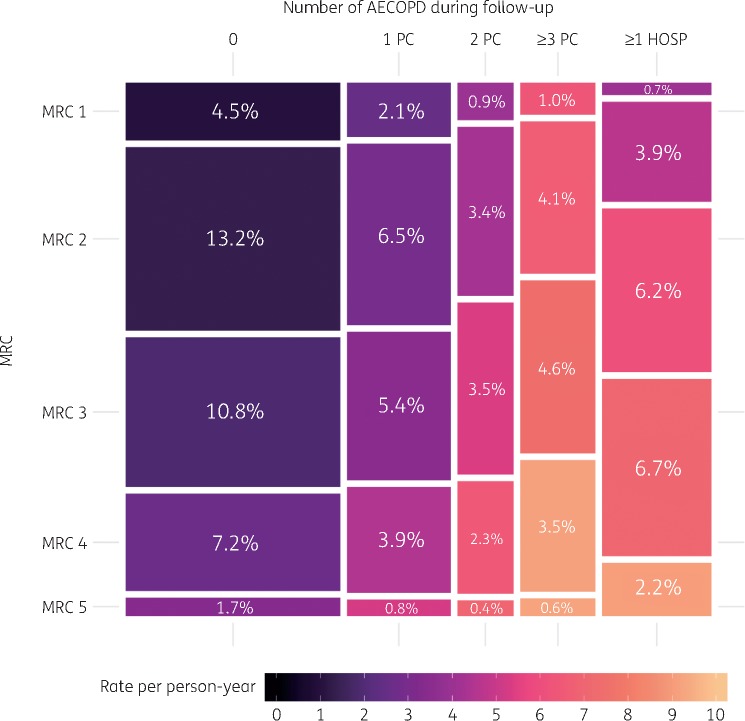
Relationship between disease severity (assessed by MRC dyspnoea scale) and the rate of antibiotic prescribing according to the number of AECOPD during follow-up (filled tiles). Tile sizes are scaled to reflect the proportion of antibiotics that were prescribed to patients in each group. For example, patients with MRC 1 and zero AECOPD during follow-up were prescribed an average of 0.97 (0.90–1.05) antibiotics per year and accounted for 4.5% of the total amount of antibiotics prescribed to patients with COPD. Rates of antibiotic prescribing for each stratum are listed in Table 3. PC, AECOPD managed in primary care; HOSP, AECOPD requiring hospitalization. Note that all rates are adjusted for age, sex, social deprivation and practice-level random effect. The rates displayed here are for a reference patient (male, aged 60–70, IMD 3). This figure appears in colour in the online version of *JAC* and in black and white in the print version of *JAC*.

Although they had the lowest rates of antibiotic prescribing (one to five prescriptions per year), patients with one or no record of AECOPD during follow-up received 56.1% of antibiotic prescribing after adjusting for age, sex and social deprivation (Figure 3; first two columns on the left). The majority of this prescribing was in patients with mild to moderate COPD (MRC 1–3), who still received 1.6 to 5.8 times as many antibiotics as the matched group without COPD. This patient group accounted for 42.5% of all antibiotic prescribing due to the large number of patients in this group (69% of all patients with COPD). At the other end of the spectrum, patients who had three or more AECOPD managed in primary care or were admitted to hospital during follow-up accounted for 33.5% of all prescriptions (Figure 3; first two columns on the right). This group had very high rates of prescribing per person, particularly 883 patients with severe COPD (MRC 4–5, 4.5% of all patients with COPD), who had an average of six to nine prescriptions per year and accounted for 13% of antibiotic prescribing. Similar results were found when stratifying by FEV1 or number of AECOPD during the baseline period (Figures S8 and S9).

## Discussion

In this large observational study, patients with COPD accounted for 11.5% of all antibiotics prescribed in primary care. Our analysis has identified two groups of COPD patients where the application of antimicrobial stewardship could have the greatest impact. Targeting patients with mild to moderate disease (GOLD 1–2, MRC 1–3 or ≤1 AECOPD in primary care) would lead to significant reductions in the amount of antibiotics that are prescribed for patients with COPD, because although these patients have comparatively low rates of antibiotic use they constitute the majority of COPD patients. However, it is also essential to target the lower number of patients with severe disease (GOLD 4, MRC 4–5 or ≥3 AECOPD in primary care or hospital admission) who have high-frequency antibiotic use, because these patients are at the greatest risk of drug resistance. Both approaches are important to ensure ongoing access to effective antibiotics.

Almost every patient with at least one AECOPD was treated with an antibiotic during follow-up and even patients without a record of AECOPD received at least twice the amount of antibiotics compared with the general population, highlighting the heavy use of antibiotics in patients with COPD. Previous studies have estimated that 60%–70% of primary care consultations for AECOPD result in an antibiotic prescription on the same day, although these studies only included prescriptions that were explicitly linked to a Read code for AECOPD.^16^^,^^17^ GPs usually have a lower threshold for prescribing antibiotics to patients with COPD, and our analysis suggests that this decision is strongly influenced by the patient’s COPD severity. Whilst measures of COPD severity such as the frequency of AECOPD in a 12 month period have been shown to predict patients’ long-term rate of AECOPD, or risk of hospitalization, their value in guiding antibiotic prescribing decisions is less clear.^18^ For example, around half of AECOPD are not caused by a bacterial infection and there is limited evidence that antibiotic treatment is effective in reducing the duration and/or severity of AECOPD, particularly for patients with mild to moderate disease in primary care. In a Cochrane review and meta-analysis of patients with AECOPD, the number needed to treat (NNT) to prevent ongoing symptoms at 1 month in patients admitted to intensive care was 3 (95% CI 2–4, high-quality evidence), which increased to 14 (95% CI 8–46) in community patients.^4^ However, when the analysis was restricted to antibiotics in current use, there was no longer evidence of benefit for patients in the community. Immediate antibiotic treatment for AECOPD in primary care may be cost-effective in preventing hospital admission or subsequent GP consultations, but this did not account for the future cost of AMR.^19^

There is a clear need to reduce the use of antibiotics in patients with COPD to mitigate the population risk of AMR and the individual’s risk of treatment failure. Patients on long-term antibiotics and those with high-frequency antibiotic use are at significant risk of resistant infection, which is augmented by frequent contact with hospital environments where drug-resistant bacteria are prevalent.^20^ In this context, the benefits of long-term antibiotic therapy to prevent exacerbations have to be balanced against the risk of promoting the emergence and spread of AMR.

Various strategies are being developed to support GPs in identifying patients who might benefit from antibiotic treatment, such as examination of sputum colour, point-of-care testing for biomarkers such as C-reactive protein or the development of risk prediction models based on data derived from EHRs.^21^^,^^22^ However, it remains difficult for GPs to objectively distinguish between bacterial and non-bacterially mediated exacerbations, making the decision to prescribe antibiotics dependent on experience and clinical judgement. Guidelines on antibiotic treatment for patients with COPD were recently updated by NICE, placing greater emphasis on antibiotic stewardship.^6^ However, it is still incumbent on GPs to judge the relative importance of factors such as the severity of symptoms, risk of hospitalization, disease severity and AMR when deciding whether to prescribe. Better information on patterns of prescribing in patients with COPD can support GPs in identifying the types of patients where it may be suitable to delay or withhold antibiotic treatment, or to consider the use of rapid diagnostic testing, as part of efforts to reduce inappropriate prescribing in primary care.

### Strengths and limitations

A strength of our approach is the use of a large, nationally representative dataset with high-quality prescribing data and the use of validated codelists to identify patients with COPD, AECOPD and comorbidities. Limitations relate to the fact that data were incomplete for exposures such as the indication for the prescription and severity measures. Increased antibiotic prescribing in high-risk groups might, for example, be linked to prescribing for suspected severe influenza. However, recording of influenza in primary care is poor and the low number of cases did not allow us to investigate this further. We did test for a confounding correlation between COPD severity and influenza vaccination but did not find any clear relationships. All analysed time periods were years with low to moderate influenza activity.^23^ Previous studies have also highlighted the limitations of using algorithms to identify patients with COPD in EHRs.^11^ We therefore selected MRC grade rather than the number of AECOPD in the previous year as the most complete method of assessing COPD severity. It is also challenging to link Read codes to episodes of antibiotic prescribing, since patients may be prescribed ‘rescue packs’ for a future rather than current AECOPD.

The analysis was limited to a single year to ensure that we had adequate sample size, to minimize seasonal effects and to ensure that the assessment of COPD severity at baseline remained valid during follow-up, since COPD severity may deteriorate over time. We replicated our analysis using data from 2013 and 2014 (Tables S14 and S15), which confirmed that our findings were consistent across different time periods. Rates of prescribing were lower in patients who were admitted to hospital compared with those with three or more AECOPD. This is likely to be because these patients received much of their care (and prescriptions) in hospital, and hospital prescribing is not captured in our dataset. Finally, CPRD records prescriptions rather than whether a drug has been dispensed, making it likely that some prescriptions are not actually taken.

### Clinical and policy implications

The need to reduce inappropriate prescribing for patients is widely acknowledged. We have identified two patient groups where the implementation of targeted antibiotic stewardship could bring the greatest reductions in total antibiotic prescribing, and minimize the risk of treatment failure in patients who are at greatest risk of AMR. The first and largest group are patients with mild to moderate disease (GOLD 1–2, MRC 1–3 or zero or one AECOPD in primary care). These individuals experience few exacerbations and have a low risk of hospital admission, but are still prescribed between one and three antibiotics per year on average. This group represents an ideal target for strategies such as delayed antibiotic prescribing. The second group are patients with very severe COPD (GOLD 4, MRC 4–5, zero to three AECOPD, or hospital admission) who are prescribed antibiotics up to 11 times per year. Whilst reducing the use of antibiotics in this latter group may be extremely challenging, new research studies are required to quantify the risk of adverse events associated with such prolonged and heavy antibiotic exposure, to ensure that these patients have ongoing access to effective antibiotic therapy.

## Supplementary Material

dkz411_Supplementary_Data
