# Supplementary material for: Opportunities to reduce antibiotic prescribing for patients with COPD in primary care: a cohort study using electronic health records from the Clinical Practice Research Datalink (CPRD)
Source: J Antimicrob Chemother. 2019 Oct 9;75(1):243–51. doi: 10.1093/jac/dkz411 (PMC6910166; doi:10.1093/jac/dkz411)
Supplement: dkz411_Supplementary_Data [file dkz411_supplementary_data.docx]

# Supplementary data

## Analysis code

The complete analysis code used for this project can be found here: <https://github.com/prockenschaub/CPRD_copd_analysis>

**Cohort selection**

**Supplementary Figure S1 - Selection criteria to define the study population**

1. **By patients at risk of having COPD**


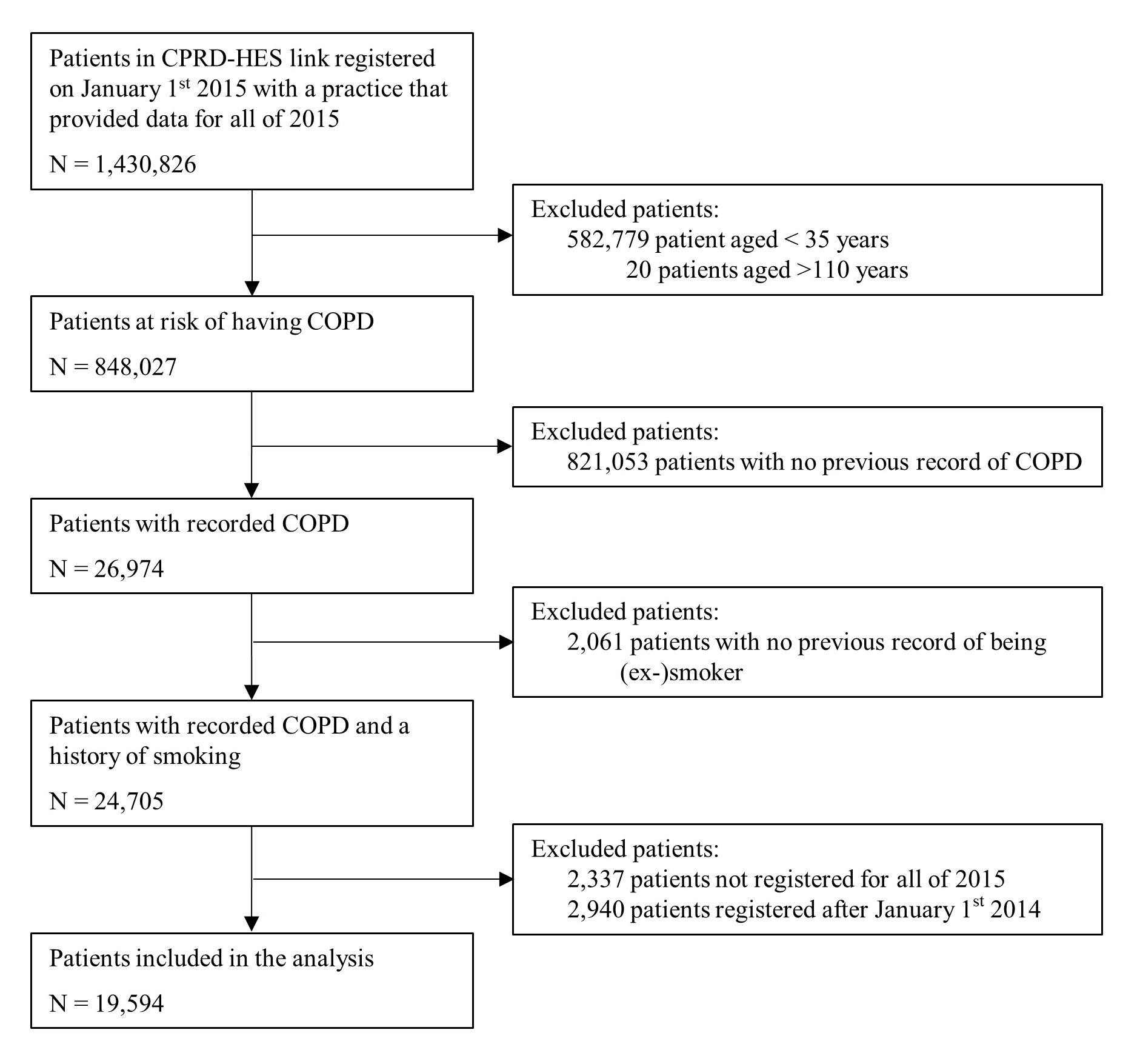


1. **By patients eligible for study inclusion**

##
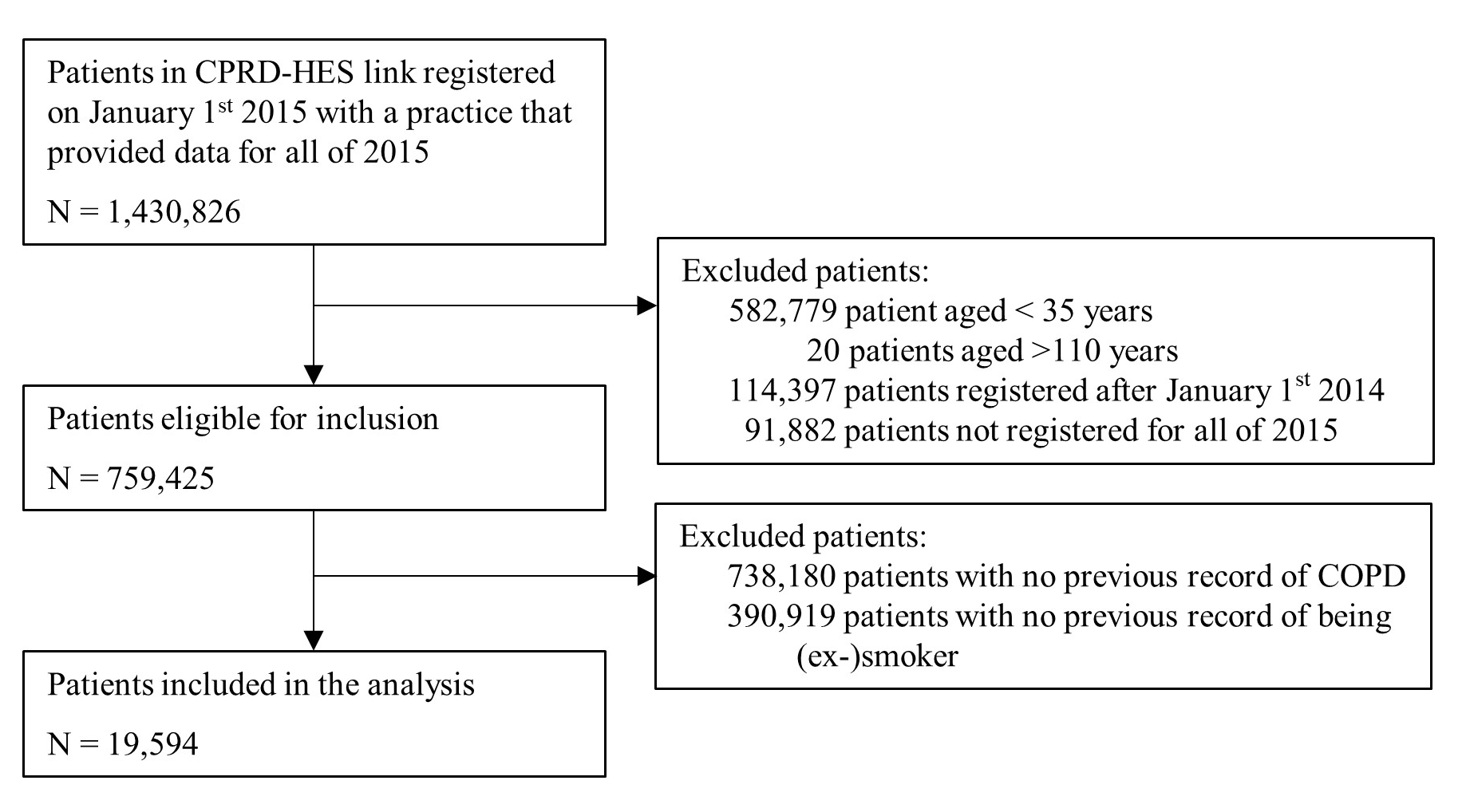


**Definition of variables**

## Definition of chronic diseases / comorbidities / lifestyle factors

Read codes used to identify the comorbidities used in this study were selected based on national primary care antibiotic prescribing guidelines. They were defined based on the Quality and Outcomes Framework v36.0.

<https://digital.nhs.uk/data-and-information/data-collections-and-data-sets/data-collections/quality-and-outcomes-framework-qof/quality-and-outcome-framework-qof-business-rules/quality-and-outcomes-framework-qof-business-rules-v36-0>

**Supplementary Table S1 - Read codes taken from the Quality and Outcomes Framework (QOF) used to identify history of chronic disease, smoking status, obesity, influenza vaccination status**

| **COPD codes** | **Description** |
| --- | --- |
| H3... | Chronic obstructive pulmonary disease |
| H31.. | Chronic bronchitis |
| H310. | Simple chronic bronchitis |
| H3100 | Chronic catarrhal bronchitis |
| H310z | Simple chronic bronchitis NOS |
| H311. | Mucopurulent chronic bronchitis |
| H3110 | Purulent chronic bronchitis |
| H3111 | Fetid chronic bronchitis |
| H311z | Mucopurulent chronic bronchitis NOS |
| H312. | Obstructive chronic bronchitis |
| H3120 | Chronic asthmatic bronchitis |
| H3121 | Emphysematous bronchitis |
| H3123 | Bronchiolitis obliterans |
| H312z | Obstructive chronic bronchitis NOS |
| H313. | Mixed simple and mucopurulent chronic bronchitis |
| H31y. | Other chronic bronchitis |
| H31y1 | Chronic tracheobronchitis |
| H31yz | Other chronic bronchitis NOS |
| H31z. | Chronic bronchitis NOS |
| H32.. | Emphysema |
| H320. | Chronic bullous emphysema |
| H3200 | Segmental bullous emphysema |
| H3201 | Zonal bullous emphysema |
| H3202 | Giant bullous emphysema |
| H3203 | Bullous emphysema with collapse |
| H320z | Chronic bullous emphysema NOS |
| H321. | Panlobular emphysema |
| H322. | Centrilobular emphysema |
| H32y. | Other emphysema |
| H32y0 | Acute vesicular emphysema |
| H32y1 | Atrophic (senile) emphysema |
| H32y2 | MacLeod's unilateral emphysema |
| H32yz | Other emphysema NOS |
| H32z. | Emphysema NOS |
| H36.. | Mild chronic obstructive pulmonary disease |
| H37.. | Moderate chronic obstructive pulmonary disease |
| H38.. | Severe chronic obstructive pulmonary disease |
| H39.. | Very severe chronic obstructive pulmonary disease |
| H3A.. | End stage chronic obstructive airways disease |
| H3B.. | Asthma-chronic obstructive pulmonary disease overlap syndrome |
| H3y.. | Other specified chronic obstructive airways disease |
| H3z.. | Chronic obstructive airways disease NOS |
| H4640 | Chronic emphysema due to chemical fumes |
| H4641 | Obliterative bronchiolitis due to chemical fumes |
| H5832 | Eosinophilic bronchitis |
| Hyu30 | [X]Other emphysema |
| Hyu31 | [X]Other specified chronic obstructive pulmonary disease |

| **Asthma codes** | **Description** |
| --- | --- |
| 173A. | Exercise induced asthma |
| H3120 | Chronic asthmatic bronchitis |
| H33.. | Asthma |
| H330. | Extrinsic (atopic) asthma |
| H3300 | Extrinsic asthma without status asthmaticus |
| H3301 | Extrinsic asthma with status asthmaticus |
| H330z | Extrinsic asthma NOS |
| H331. | Intrinsic asthma |
| H3310 | Intrinsic asthma without status asthmaticus |
| H3311 | Intrinsic asthma with status asthmaticus |
| H331z | Intrinsic asthma NOS |
| H332. | Mixed asthma |
| H334. | Brittle asthma |
| H335. | Chronic asthma with fixed airflow obstruction |
| H33z. | Asthma unspecified |
| H33z0 | Status asthmaticus NOS |
| H33z1 | Asthma attack |
| H33z2 | Late-onset asthma |
| H33zz | Asthma NOS |
| H3B.. | Asthma-chronic obstructive pulmonary disease overlap syndrome |

| **CHD codes** | **Description** |
| --- | --- |
| G3...00 | Ischaemic heart disease |
| G30..00 | Acute myocardial infarction |
| G300.00 | Acute anterolateral infarction |
| G301.00 | Other specified anterior myocardial infarction |
| G301000 | Acute anteroapical infarction |
| G301100 | Acute anteroseptal infarction |
| G301z00 | Anterior myocardial infarction NOS |
| G302.00 | Acute inferolateral infarction |
| G303.00 | Acute inferoposterior infarction |
| G304.00 | Posterior myocardial infarction NOS |
| G305.00 | Lateral myocardial infarction NOS |
| G306.00 | True posterior myocardial infarction |
| G307.00 | Acute subendocardial infarction |
| G307000 | Acute non-Q wave infarction |
| G307100 | Acute non-ST segment elevation myocardial infarction |
| G308.00 | Inferior myocardial infarction NOS |
| G309.00 | Acute Q-wave infarct |
| G30B.00 | Acute posterolateral myocardial infarction |
| G30X.00 | Acute transmural myocardial infarction of unspecified site |
| G30X000 | Acute ST segment elevation myocardial infarction |
| G30y.00 | Other acute myocardial infarction |
| G30y000 | Acute atrial infarction |
| G30y100 | Acute papillary muscle infarction |
| G30y200 | Acute septal infarction |
| G30yz00 | Other acute myocardial infarction NOS |
| G30z.00 | Acute myocardial infarction NOS |
| G31..00 | Other acute and subacute ischaemic heart disease |
| G311.00 | Preinfarction syndrome |
| G311000 | Myocardial infarction aborted |
| G311100 | Unstable angina |
| G311200 | Angina at rest |
| G311300 | Refractory angina |
| G311400 | Worsening angina |
| G311500 | Acute coronary syndrome |
| G311z00 | Preinfarction syndrome NOS |
| G312.00 | Coronary thrombosis not resulting in myocardial infarction |
| G31y.00 | Other acute and subacute ischaemic heart disease |
| G31y000 | Acute coronary insufficiency |
| G31y100 | Microinfarction of heart |
| G31y200 | Subendocardial ischaemia |
| G31y300 | Transient myocardial ischaemia |
| G31yz00 | Other acute and subacute ischaemic heart disease NOS |
| G32..00 | Old myocardial infarction |
| G33..00 | Angina pectoris |
| G330.00 | Angina decubitus |
| G330000 | Nocturnal angina |
| G330z00 | Angina decubitus NOS |
| G33z.00 | Angina pectoris NOS |
| G33z000 | Status anginosus |
| G33z100 | Stenocardia |
| G33z200 | Syncope anginosa |
| G33z300 | Angina on effort |
| G33z400 | Ischaemic chest pain |
| G33z500 | Post infarct angina |
| G33z600 | New onset angina |
| G33z700 | Stable angina |
| G33zz00 | Angina pectoris NOS |
| G34..00 | Other chronic ischaemic heart disease |
| G340.00 | Coronary atherosclerosis |
| G340000 | Single coronary vessel disease |
| G340100 | Double coronary vessel disease |
| G342.00 | Atherosclerotic cardiovascular disease |
| G343.00 | Ischaemic cardiomyopathy |
| G344.00 | Silent myocardial ischaemia |
| G34y.00 | Other specified chronic ischaemic heart disease |
| G34y000 | Chronic coronary insufficiency |
| G34y100 | Chronic myocardial ischaemia |
| G34yz00 | Other specified chronic ischaemic heart disease NOS |
| G34z.00 | Other chronic ischaemic heart disease NOS |
| G34z000 | Asymptomatic coronary heart disease |
| G35..00 | Subsequent myocardial infarction |
| G350.00 | Subsequent myocardial infarction of anterior wall |
| G351.00 | Subsequent myocardial infarction of inferior wall |
| G353.00 | Subsequent myocardial infarction of other sites |
| G35X.00 | Subsequent myocardial infarction of unspecified site |
| G38..00 | Postoperative myocardial infarction |
| G380.00 | Postoperative transmural myocardial infarction of anterior wall |
| G381.00 | Postoperative transmural myocardial infarction of inferior wall |
| G382.00 | Postoperative transmural myocardial infarction of other sites |
| G383.00 | Postoperative transmural myocardial infarction of unspecified site |
| G384.00 | Postoperative subendocardial myocardial infarction |
| G38z.00 | Postoperative myocardial infarction, unspecified |
| G39..00 | Coronary microvascular disease |
| G3y..00 | Other specified ischaemic heart disease |
| G3z..00 | Ischaemic heart disease NOS |
| Gyu3.00 | [X]Ischaemic heart diseases |
| Gyu3000 | [X]Other forms of angina pectoris |
| Gyu3200 | [X]Other forms of acute ischaemic heart disease |
| Gyu3300 | [X]Other forms of chronic ischaemic heart disease |
| Gyu3400 | [X]Acute transmural myocardial infarction of unspecified site |
| Gyu3500 | [X]Subsequent myocardial infarction of other sites |
| Gyu3600 | [X]Subsequent myocardial infarction of unspecified site |

| **CKD codes** | **Description** |
| --- | --- |
| 1Z12. | Chronic kidney disease stage 3 |
| 1Z13. | Chronic kidney disease stage 4 |
| 1Z14. | Chronic kidney disease stage 5 |
| 1Z15. | Chronic kidney disease stage 3A |
| 1Z16. | Chronic kidney disease stage 3B |
| 1Z1a. | CKD G4A1 - chronic kidney disease with glomerular filtration rate category G4 and albuminuria category A1 |
| 1Z1b. | CKD G4A2 - chronic kidney disease with glomerular filtration rate category G4 and albuminuria category A2 |
| 1Z1B. | Chronic kidney disease stage 3 with proteinuria |
| 1Z1c. | CKD G4A3 - chronic kidney disease with glomerular filtration rate category G4 and albuminuria category A3 |
| 1Z1C. | Chronic kidney disease stage 3 without proteinuria |
| 1Z1d. | CKD G5A1 - chronic kidney disease with glomerular filtration rate category G5 and albuminuria category A1 |
| 1Z1D. | Chronic kidney disease stage 3A with proteinuria |
| 1Z1e. | CKD G5A2 - chronic kidney disease with glomerular filtration rate category G5 and albuminuria category A2 |
| 1Z1E. | Chronic kidney disease stage 3A without proteinuria |
| 1Z1f. | CKD G5A3 - chronic kidney disease with glomerular filtration rate category G5 and albuminuria category A3 |
| 1Z1F. | Chronic kidney disease stage 3B with proteinuria |
| 1Z1G. | Chronic kidney disease stage 3B without proteinuria |
| 1Z1H. | Chronic kidney disease stage 4 with proteinuria |
| 1Z1J. | Chronic kidney disease stage 4 without proteinuria |
| 1Z1K. | Chronic kidney disease stage 5 with proteinuria |
| 1Z1L. | Chronic kidney disease stage 5 without proteinuria |
| 1Z1T. | CKD G3aA1 - chronic kidney disease with glomerular filtration rate category G3a and albuminuria category A1 |
| 1Z1V. | CKD G3aA2 - chronic kidney disease with glomerular filtration rate category G3a and albuminuria category A2 |
| 1Z1W. | CKD G3aA3 - chronic kidney disease with glomerular filtration rate category G3a and albuminuria category A3 |
| 1Z1X. | CKD G3bA1 - chronic kidney disease with glomerular filtration rate category G3b and albuminuria category A1 |
| 1Z1Y. | CKD G3bA2 - chronic kidney disease with glomerular filtration rate category G3b and albuminuria category A2 |
| 1Z1Z. | CKD G3bA3 - chronic kidney disease with glomerular filtration rate category G3b and albuminuria category A3 |
| K053. | Chronic kidney disease stage 3 |
| K054. | Chronic kidney disease stage 4 |
| K055. | Chronic kidney disease stage 5 |

| **Diabetes codes** | **Description** |
| --- | --- |
| C10.. | Diabetes mellitus |
| C109J | Insulin treated Type 2 diabetes mellitus |
| C109K | Hyperosmolar non-ketotic state in type 2 diabetes mellitus |
| C10C. | Diabetes mellitus autosomal dominant |
| C10D. | Diabetes mellitus autosomal dominant type 2 |
| C10E. | Type 1 diabetes mellitus |
| C10E0 | Type 1 diabetes mellitus with renal complications |
| C10E1 | Type 1 diabetes mellitus with ophthalmic complications |
| C10E2 | Type 1 diabetes mellitus with neurological complications |
| C10E3 | Type 1 diabetes mellitus with multiple complications |
| C10E4 | Unstable type 1 diabetes mellitus |
| C10E5 | Type 1 diabetes mellitus with ulcer |
| C10E6 | Type 1 diabetes mellitus with gangrene |
| C10E7 | Type 1 diabetes mellitus with retinopathy |
| C10E8 | Type 1 diabetes mellitus - poor control |
| C10E9 | Type 1 diabetes mellitus maturity onset |
| C10EA | Type 1 diabetes mellitus without complication |
| C10EB | Type 1 diabetes mellitus with mononeuropathy |
| C10EC | Type 1 diabetes mellitus with polyneuropathy |
| C10ED | Type 1 diabetes mellitus with nephropathy |
| C10EE | Type 1 diabetes mellitus with hypoglycaemic coma |
| C10EF | Type 1 diabetes mellitus with diabetic cataract |
| C10EG | Type 1 diabetes mellitus with peripheral angiopathy |
| C10EH | Type 1 diabetes mellitus with arthropathy |
| C10EJ | Type 1 diabetes mellitus with neuropathic arthropathy |
| C10EK | Type 1 diabetes mellitus with persistent proteinuria |
| C10EL | Type 1 diabetes mellitus with persistent microalbuminuria |
| C10EM | Type 1 diabetes mellitus with ketoacidosis |
| C10EN | Type 1 diabetes mellitus with ketoacidotic coma |
| C10EP | Type 1 diabetes mellitus with exudative maculopathy |
| C10EQ | Type 1 diabetes mellitus with gastroparesis |
| C10ER | Latent autoimmune diabetes mellitus in adult |
| C10F. | Type 2 diabetes mellitus |
| C10F0 | Type 2 diabetes mellitus with renal complications |
| C10F1 | Type 2 diabetes mellitus with ophthalmic complications |
| C10F2 | Type 2 diabetes mellitus with neurological complications |
| C10F3 | Type 2 diabetes mellitus with multiple complications |
| C10F4 | Type 2 diabetes mellitus with ulcer |
| C10F5 | Type 2 diabetes mellitus with gangrene |
| C10F6 | Type 2 diabetes mellitus with retinopathy |
| C10F7 | Type 2 diabetes mellitus - poor control |
| C10F9 | Type 2 diabetes mellitus without complication |
| C10FA | Type 2 diabetes mellitus with mononeuropathy |
| C10FB | Type 2 diabetes mellitus with polyneuropathy |
| C10FC | Type 2 diabetes mellitus with nephropathy |
| C10FD | Type 2 diabetes mellitus with hypoglycaemic coma |
| C10FE | Type 2 diabetes mellitus with diabetic cataract |
| C10FF | Type 2 diabetes mellitus with peripheral angiopathy |
| C10FG | Type 2 diabetes mellitus with arthropathy |
| C10FH | Type 2 diabetes mellitus with neuropathic arthropathy |
| C10FJ | Insulin treated Type 2 diabetes mellitus |
| C10FK | Hyperosmolar non-ketotic state in type 2 diabetes mellitus |
| C10FL | Type 2 diabetes mellitus with persistent proteinuria |
| C10FM | Type 2 diabetes mellitus with persistent microalbuminuria |
| C10FN | Type 2 diabetes mellitus with ketoacidosis |
| C10FP | Type 2 diabetes mellitus with ketoacidotic coma |
| C10FQ | Type 2 diabetes mellitus with exudative maculopathy |
| C10FR | Type 2 diabetes mellitus with gastroparesis |
| C10FS | Maternally inherited diabetes mellitus |
| C10G. | Secondary pancreatic diabetes mellitus |
| C10G0 | Secondary pancreatic diabetes mellitus without complication |
| C10H. | Diabetes mellitus induced by non-steroid drugs |
| C10H0 | Diabetes mellitus induced by non-steroid drugs without complication |
| C10M. | Lipoatrophic diabetes mellitus |
| C10M0 | Lipoatrophic diabetes mellitus without complication |
| C10N. | Secondary diabetes mellitus |
| C10N0 | Secondary diabetes mellitus without complication |
| C10N1 | Cystic fibrosis related diabetes mellitus |
| C10P. | Diabetes mellitus in remission |
| C10P0 | Type I diabetes mellitus in remission |
| C10P1 | Type II diabetes mellitus in remission |
| C10Q. | Maturity onset diabetes of the young type 5 |
| PKyP. | Diabetes insipidus, diabetes mellitus, optic atrophy and deafness |

| **Heart failure codes** | **Description** |
| --- | --- |
| 662f. | New York Heart Association classification - class I |
| 662g. | New York Heart Association classification - class II |
| 662h. | New York Heart Association classification - class III |
| 662i. | New York Heart Association classification - class IV |
| G1yz1 | Rheumatic left ventricular failure |
| G58.. | Heart failure |
| G580. | Congestive heart failure |
| G5800 | Acute congestive heart failure |
| G5801 | Chronic congestive heart failure |
| G5802 | Decompensated cardiac failure |
| G5803 | Compensated cardiac failure |
| G5804 | Congestive heart failure due to valvular disease |
| G581. | Left ventricular failure |
| G5810 | Acute left ventricular failure |
| G582. | Acute heart failure |
| G583. | Heart failure with normal ejection fraction |
| G584. | Right ventricular failure |
| G58z. | Heart failure NOS |

| **Obesity codes** | **Description** |
| --- | --- |
| 22K5. | Body mass index 30+ - obesity |
| 22K7. | Body mass index 40+ - severely obese |
| 22KC. | Obese class I (body mass index 30.0 - 34.9) |
| 22KD. | Obese class II (body mass index 35.0 - 39.9) |
| 22KE. | Obese class III (body mass index equal to or greater than 40.0) |
| 22K.. | Body Mass Index |

| **PAD codes** | **Description** |
| --- | --- |
| G73.. | Other peripheral vascular disease |
| G734. | Peripheral arterial disease |
| G73y. | Other specified peripheral vascular disease |
| G73z. | Peripheral vascular disease NOS |
| G73z0 | Intermittent claudication |
| G73zz | Peripheral vascular disease NOS |
| Gyu74 | [X]Other specified peripheral vascular diseases |

| **Smoking codes** | **Description** |
| --- | --- |
| 137.. | Tobacco consumption |
| 1371. | Never smoked tobacco |
| 1372. | Trivial smoker - < 1 cig/day |
| 1373. | Light smoker - 1-9 cigs/day |
| 1374. | Moderate smoker - 10-19 cigs/d |
| 1375. | Heavy smoker - 20-39 cigs/day |
| 1376. | Very heavy smoker - 40+cigs/d |
| 1377. | Ex-trivial smoker (<1/day) |
| 1378. | Ex-light smoker (1-9/day) |
| 1379. | Ex-moderate smoker (10-19/day) |
| 137a. | Pipe tobacco consumption |
| 137A. | Ex-heavy smoker (20-39/day) |
| 137b. | Ready to stop smoking |
| 137B. | Ex-very heavy smoker (40+/day) |
| 137c. | Thinking about stopping smoking |
| 137C. | Keeps trying to stop smoking |
| 137d. | Not interested in stopping smoking |
| 137D. | Admitted tobacco cons untrue ? |
| 137e. | Smoking restarted |
| 137f. | Reason for restarting smoking |
| 137F. | Ex-smoker - amount unknown |
| 137G. | Trying to give up smoking |
| 137h. | Minutes from waking to first tobacco consumption |
| 137H. | Pipe smoker |
| 137J. | Cigar smoker |
| 137j. | Ex-cigarette smoker |
| 137K. | Stopped smoking |
| 137l. | Ex roll-up cigarette smoker |
| 137m. | Failed attempt to stop smoking |
| 137M. | Rolls own cigarettes |
| 137N. | Ex pipe smoker |
| 137o. | Waterpipe tobacco consumption |
| 137O. | Ex cigar smoker |
| 137P. | Cigarette smoker |
| 137Q. | Smoking started |
| 137R. | Current smoker |
| 137S. | Ex smoker |
| 137T. | Date ceased smoking |
| 137V. | Smoking reduced |
| 137X. | Cigarette consumption |
| 137Y. | Cigar consumption |
| 137Z. | Tobacco consumption NOS |
| 13p50 | Practice based smoking cessation programme start date |
| 8CAL. | Smoking cessation advice |
| 8H7i. | Referral to smoking cessation advisor |
| 8HkQ. | Referral to NHS stop smoking service |
| 8HTK. | Referral to stop-smoking clinic |
| 8IAj. | Smoking cessation advice declined |
| 8IEK. | Smoking cessation programme declined |
| 8IEo. | Referral to smoking cessation service declined |
| 8T08. | Referral to smoking cessation service |
| 9N2k. | Seen by smoking cessation advisor |
| 9Ndf. | Consent given for follow-up by smoking cessation team |
| 9Ndg. | Declined consent for follow-up by smoking cessation team |

| **Stroke codes** | **Description** |
| --- | --- |
| Fyu55 | [X]Other transient cerebral ischaemic attacks and related syndromes |
| G61.. | Intracerebral haemorrhage |
| G610. | Cortical haemorrhage |
| G611. | Internal capsule haemorrhage |
| G612. | Basal nucleus haemorrhage |
| G613. | Cerebellar haemorrhage |
| G614. | Pontine haemorrhage |
| G615. | Bulbar haemorrhage |
| G616. | External capsule haemorrhage |
| G618. | Intracerebral haemorrhage, multiple localized |
| G619. | Lobar cerebral haemorrhage |
| G61X. | Intracerebral haemorrhage in hemisphere, unspecified |
| G61X0 | Left sided intracerebral haemorrhage, unspecified |
| G61X1 | Right sided intracerebral haemorrhage, unspecified |
| G61z. | Intracerebral haemorrhage NOS |
| G63y0 | Cerebral infarct due to thrombosis of precerebral arteries |
| G63y1 | Cerebral infarction due to embolism of precerebral arteries |
| G64.. | Cerebral arterial occlusion |
| G640. | Cerebral thrombosis |
| G6400 | Cerebral infarction due to thrombosis of cerebral arteries |
| G641. | Cerebral embolism |
| G6410 | Cerebral infarction due to embolism of cerebral arteries |
| G64z. | Cerebral infarction NOS |
| G64z0 | Brainstem infarction |
| G64z1 | Wallenberg syndrome |
| G64z2 | Left sided cerebral infarction |
| G64z3 | Right sided cerebral infarction |
| G64z4 | Infarction of basal ganglia |
| G65.. | Transient cerebral ischaemia |
| G650. | Basilar artery syndrome |
| G651. | Vertebral artery syndrome |
| G6510 | Vertebro-basilar artery syndrome |
| G652. | Subclavian steal syndrome |
| G653. | Carotid artery syndrome hemispheric |
| G654. | Multiple and bilateral precerebral artery syndromes |
| G656. | Vertebrobasilar insufficiency |
| G657. | Carotid territory transient ischaemic attack |
| G65y. | Other transient cerebral ischaemia |
| G65z. | Transient cerebral ischaemia NOS |
| G65z0 | Impending cerebral ischaemia |
| G65z1 | Intermittent cerebral ischaemia |
| G65zz | Transient cerebral ischaemia NOS |
| G66.. | Stroke and cerebrovascular accident unspecified |
| G660. | Middle cerebral artery syndrome |
| G661. | Anterior cerebral artery syndrome |
| G662. | Posterior cerebral artery syndrome |
| G663. | Brain stem stroke syndrome |
| G664. | Cerebellar stroke syndrome |
| G665. | Pure motor lacunar syndrome |
| G666. | Pure sensory lacunar syndrome |
| G667. | Left sided CVA |
| G668. | Right sided CVA |
| G6760 | Cerebral infarction due to cerebral venous thrombosis, nonpyogenic |
| G6W.. | Cerebral infarction due to unspecified occlusion or stenosis of precerebral arteries |
| G6X.. | Cerebral infarction due to unspecified occlusion or stenosis of cerebral arteries |
| Gyu62 | [X]Other intracerebral haemorrhage |
| Gyu63 | [X]Cerebral infarction due to unspecified occlusion or stenosis of cerebral arteries |
| Gyu64 | [X]Other cerebral infarction |
| Gyu65 | [X]Occlusion and stenosis of other precerebral arteries |
| Gyu66 | [X]Occlusion and stenosis of other cerebral arteries |
| Gyu6F | [X]Intracerebral haemorrhage in hemisphere, unspecified |
| Gyu6G | [X]Cerebral infarction due to unspecified occlusion or stenosis of precerebral arteries |
| ZV12D | [V]Personal history of transient ischaemic attack |

| **Flu vacc. codes** | **Description** |
| --- | --- |
| 65E2000 | Seasonal influenza vaccin given by other healthcare provider |
| 65E2100 | First intranasal seasonal flu vacc gvn by othr hlthcare prov |
| 65E2200 | Secnd intranasal seasonal flu vacc gvn by othr hlthcare prov |
| 65E2300 | 2nd intramuscular seasonal influenza vacc given by other HCP |
| 65E2400 | 1st intramuscular seasonal influenza vacc given by other HCP |
| 65ED.00 | Seasonal influenza vaccination |
| 65ED000 | Seasonal influenza vaccination given by pharmacist |
| 65ED100 | Administration of first intranasal seasonal influenza vacc |
| 65ED200 | Seasonal influenza vaccination given while hospital inpt |
| 65ED300 | Administration of second intranasal seasonal influenza vacc |
| 65ED400 | Administration of first inactivated seasonal influenza vacc |

## Definition of FEV1 as percent of predicted

Measures of forced expiratory volume in 1 second as percent of predicted (FEV1-%) were identified using Read codes covered within the COPD guidance of the Quality and Outcomes Framework v36.0.

<https://digital.nhs.uk/data-and-information/data-collections-and-data-sets/data-collections/quality-and-outcomes-framework-qof/quality-and-outcome-framework-qof-business-rules/quality-and-outcomes-framework-qof-business-rules-v36-0>

**Supplementary Table S2 - Read codes adapted from the Quality and Outcomes Framework (QOF) used to identify measurements of forced expiratory volume in 1 second (FEV1) and forced vital capacity (FVC)**

| **FEV1/FVC codes** | **Description** | **Type** |
| --- | --- | --- |
| 3396.00 | Forced vital capacity - FVC | fvc |
| 3396000 | FVC - forced vital capacity normal | fvc |
| 3396100 | FVC - forced vital capacity abnormal | fvc |
| 3396300 | FVC after change of bronchodilator | fvc |
| 3397.00 | Forced expiratory volume - FEV | fev |
| 3397000 | FEV normal | fev |
| 3397200 | FEV1 after change of bronchodilator | fev |
| 3398.00 | FEV1/FVC ratio normal | fev/fvc |
| 3399.00 | FEV1/FVC ratio abnormal | fev/fvc |
| 339a.00 | FEV1 before bronchodilation | fev |
| 339b.00 | FEV1 after bronchodilation | fev |
| 339e.00 | FEV1 pre steroids | fev |
| 339f.00 | FEV1 post steroids | fev |
| 339h.00 | FVC after bronchodilation | fvc |
| 339j.00 | FEV1/FVC ratio pre steroids | fev/fvc |
| 339k.00 | FEV1/FVC ratio post steroids | fev/fvc |
| 339l.00 | FEV1/FVC ratio before bronchodilator | fev/fvc |
| 339m.00 | FEV1/FVC ratio after bronchodilator | fev/fvc |
| 339M.00 | FEV1/FVC ratio | fev/fvc |
| 339O.00 | Forced expired volume in 1 second | fev |
| 339O100 | Forced expired volume in one second/vital capacity ratio | fev/fvc |
| 339R.00 | FEV1/FVC percent | fev/fvc |
| 339r.00 | FEV1/VC percent | fev/fvc |
| 339s.00 | Forced vital capacity before bronchodilation | fvc |
| 339S.00 | Percent predicted FEV1 | predict fev |
| 339S000 | Percentage predicted FEV1 after bronchodilation | predict fev |

Codes used to define FEV1-% fell within three broad classes: a directly recorded measure of FEV1-%, a measure of absolute FEV1 (i.e. **not** as percent of predicted but as L/second), a measure of the ratio of FEV1 to total forced vital capacity (FVC), and a measure of absolute FVC. No attempt was made to distinguish between pre and post steroids/bronchodilator measurements.

Using records for these four types of measurement, we attempted to calculate a plausible estimate of FEV1-% for each patient and date at which there was at least on recorded value. The following hierarchical approach was used to estimate FEV1-%:

**Supplementary Table S3 - Algorithm used to calculate forced expiratory volume in 1 second as % of predicted (FEV1-%)**

| **#** | **Rule** | **FEV1-%** |
| --- | --- | --- |
| 1 | If Read code indicated FEV1-% | Recorded value |
| 2 | If Read code indicated absolute FEV1 in litres and recorded value was between 0.1 and 7 | Recorded value divided by the predicted FEV1 of the patient |
| 3 | If Read code indicated both a FEV1/FVC in % between 7 and 100 and an absolute FVC in litres between 0.1 and 7 | Recorded value of FEV1/FVC multiplied by recorded value of FVC divided by the predicted FEV1 of the patient |

All estimated measures of FEV1 were further constrained to lie within a reasonable range of 5 to 100. Measures above 100 are unlikely for true COPD patients. Measures below 5 are biologically implausible and indicate a mislabelling of litres as %. Estimates outside this range were set to missing.

## Definition of MRC breathlessness score

Self-reported MRC breathlessness scores were identified using the appropriate Read codes.

**Supplementary Table S4 - Read codes taken from the Quality and Outcomes Framework (QOF) used to identify measurements of the MRC breathlessness score**

| **MRC codes** | **Description** |
| --- | --- |
| 173H.00 | MRC Breathlessness Scale: grade 1 |
| 173I.00 | MRC Breathlessness Scale: grade 2 |
| 173J.00 | MRC Breathlessness Scale: grade 3 |
| 173K.00 | MRC Breathlessness Scale: grade 4 |
| 173L.00 | MRC Breathlessness Scale: grade 5 |

## Definition of acute exacerbations of COPD

Acute exacerbations of COPD (AECOPD) in primary and secondary care were identified following peer-reviewed algorithms developed and validated for CPRD-HES.[^3,4^](https://paperpile.com/c/QI1HRr/qIjZ+Ch3d) AECOPDs were considered moderate if they were entirely treated in primary care and as severe if they required hospitalisation. Adapting a classification published by the same author group that developed the algorithms[^5^](https://paperpile.com/c/QI1HRr/Jpz6), patients were grouped into those with no exacerbations, one moderate exacerbation, two moderate exacerbations, three or more moderate exacerbations, and one or more severe exacerbations (irrespective of the number of additional moderate exacerbations).

Moderate AECOPD were identified using the algorithms 3, 5, 6, 8 and 12 specified by Rothnie et al. 2016. [^4^](https://paperpile.com/c/QI1HRr/Ch3d) Together, these algorithms were shown to have a positive predictive value of 85.5 and a sensitivity of 62.9, and they were chosen as they exhibited a balance between PPV and sensitivity. The exact algorithm is defined in Supplementary Table 6.

**Supplementary Table S5 - Algorithm used to identify moderate acute exacerbations of chronic obstructive pulmonary disease**

| **#** | **Algorithm** | **Notes/Comments** |
| --- | --- | --- |
| 3 | Oral corticosteroids and antibiotic prescription | For 5-14 days, both on the same day |
| 5 | Exacerbation Symptom definition and oral corticosteroid prescription | Codes suggesting increase in two or more of: breathlessness, cough, or sputum volume and/or purulence. Medical codes must have been on the same day as prescription. Duration of prescription was not limited. |
| 6 | Exacerbation Symptom definition and antibiotic prescription | Codes suggesting increase in two or more of: breathlessness, cough, or sputum volume and/or purulence. Medical codes must have been on the same day as prescription. Duration of prescription was not limited. |
| 8 | Lower respiratory tract infection (LRTI) code | Specifically excluding codes for pneumonia |
| 12 | AECOPD code |  |

Table copied from Rothnie et al. 2016. See original source for the complete definitions of all 15 algorithms. Code lists for the above definitions are available in the supplementary material of the original source.

Severe AECOPD was defined as an admission to hospital due to an exacerbation. We used algorithm 5 as defined in Rothnie et al. 2016.[^3^](https://paperpile.com/c/QI1HRr/qIjZ) This algorithm defined a hospitalisation for AECOPD as a inpatient stay with an ICD-10 discharge code for AECOPD (J44.0 and J44.1) or lower respiratory tract infection (J22) in any position. We did not include stays with a general COPD code (J44.9) in the first position in our definition, as our data did not contain the position of the recorded ICD-10 code and using a general COPD code in any position was shown to dramatically overestimate the number of severe AECOPD.

## Definition of patterns of antibiotic prescribing

Prescriptions were categorised as initial prescribing, re-prescribing for the same indication, continuous prescribing that lasted for less than 6 months, and continuous prescribing of 6 months or longer. The definitions used in this paper were adapted from those used previously by researchers from Public Health England (Dolk et al. 2018).

A prescription was considered to be an initial prescription if there was no primary care antibiotic prescription for the same body system in the 30 days prior (see *Definition of indication for prescribing* below for an in-depth description of how body systems were defined). In contrast, re-prescribing were all prescriptions that followed a previous prescription for the same indication in the prior 30 days. The same indication for the purpose of identifying re-prescribing was defined as a) any antibiotic prescription for the same recorded body system or b) the same antibiotic without contradicting recorded body systems (i.e. at least one of the prescriptions had no recorded indication).

Both initial prescriptions and re-prescribing were reclassified as continuous prescribing if any of the following conditions were fulfilled: a) prescribing was explicitly labelled as being part of a prescribing sequence (CPRD field *issueseq*), or b) the initial or re-prescribing was part of a sequence of three or more prescriptions of the same antibiotic or for the same indication within 30 days between each consecutive prescription. If the prescribing sequence covered 162 out of 180 days or if there was at least one prescription each month for 6 months, continuous prescribing was said to be 6 months or longer. Otherwise, the continuous prescribing was labelled less than 6 months.

## Definition of indication for prescribing

In order to assign an indication for prescribing to as many antibiotic prescriptions as possible, all diagnostic Read codes recorded on the same calendar day as an antibiotic prescription were extracted from CPRD. Using a code list previously published by researchers from Public Health England (Dolk et al. 2018), we filtered these codes for Read codes potentially related to antibiotic prescribing and collapsed them into the following body systems and clinical conditions:

- Respiratory tract and Ear/Nose/Throat (ENT)
  - COPD related (including lower respiratory tract)
  - Cough
  - Upper respiratory tract infection (including sore throat)
  - Other respiratory tract conditions
- Urogenital tract
- Skin and soft tissue (excluding wounds/bites)
- Other body systems
  - Wounds/bites
  - Gastrointestinal
  - Central nervous system
  - Eye
  - Cardiovascular
  - Dental/mouth
  - Musculoskeletal
  - Miscellaneous
  - Multiple body systems

Following the original approach used by Dolk et al., codes were further grouped into those describing diagnoses (e.g. H312200 - Acute exacerbation of chronic obstructive airways disease) and symptoms (e.g. 171..00 - Cough). If more than one candidate code was recorded for an antibiotic prescription, precedence was given to diagnoses. If two or more codes (both diagnoses or both symptoms and no diagnosis) for different body systems remained, the indication was labelled as having multiple indicated body systems.

For all prescriptions, the most likely indication for prescribing was assigned based on the following sequential rules, using the first that applied:

**Supplementary Table S6 - Stepwise algorithm used to define indication for antibiotic prescribing**

| **#** | **Rule** | **Indication** |
| --- | --- | --- |
| 1 | If prescription was Nitrofurantoin | Urogenital tract |
| 2 | If validated acute exacerbation of COPD recorded on the same day | Respiratory tract and ENT / COPD related |
| 3 | If another relevant Read code was recorded on the same day | Body system/condition recorded on the day (as defined above) |
| 4 | If a relevant Read code was recorded previously and both records shared the same consultation identifier | Body system/condition recorded on the previous day |
| 5 | If the prescription was part of a prescribing sequence | Body system/condition recorded on the same day as the start of the sequence |
| 6 | If a relevant Read code was recorded on the same day as a prescription of the same antibiotic in the previous 30 days | Body system/condition recorded on the same day as the previous prescription |
| 7 | If a relevant Read code was recorded within the previous 7 days | Body system/condition recorded previously irrespective of prescription |
| 8 | If prescribing was part of a continuous prescribing sequence of >6 months and was either   - Trimethoprim: ≤ 100mg/day - Azithromycin: ≤ 250mg/day | Trimethoprim: Urinary tract  Azithromycin: Respiratory tract and ENT / COPD related |

Rules were adapted from the most sensitive algorithm proposed by Dolk et al.

**Missing data and multiple imputation**

There was missing information for forced expiratory volume in one second (FEV1) and for the MRC breathlessness score. For both measures, the latest record within 1 year prior to entry date was chosen for each patient, as Quality and Outcome Framework (QOF) v36.0 targets for COPD include a yearly assessment of both (Indicators COPD003 and COPD004). Missing information on both variables was imputed via multiple imputation using the R package mice (multivariate imputation by chained equations - version 3.3.0).

## Proportion of information missing

There were 7,578 (38.7%) patients without a recent measurement of FEV1 (Supplementary Figure 4) and 5,044 (25.7%) patients without a recent measurement of MRC score (Supplementary Figure 5). However, 96% of patients had at least one measure of FEV1 or MRC within 4 years prior to study entry (Table 1). In patients that had multiple measurements of either measure, subsequent measures of FEV1 and MRC were fairly well correlated with values at baseline (Supplementary Figure 6).

**Supplementary Figure 2 - The cumulative proportion of patients with a valid measurement of FEV1 as a function of the observation time. The blue line represents the proportion of patients with a valid measurement within one year prior to study entry (January 1st - December 31st 2014)**


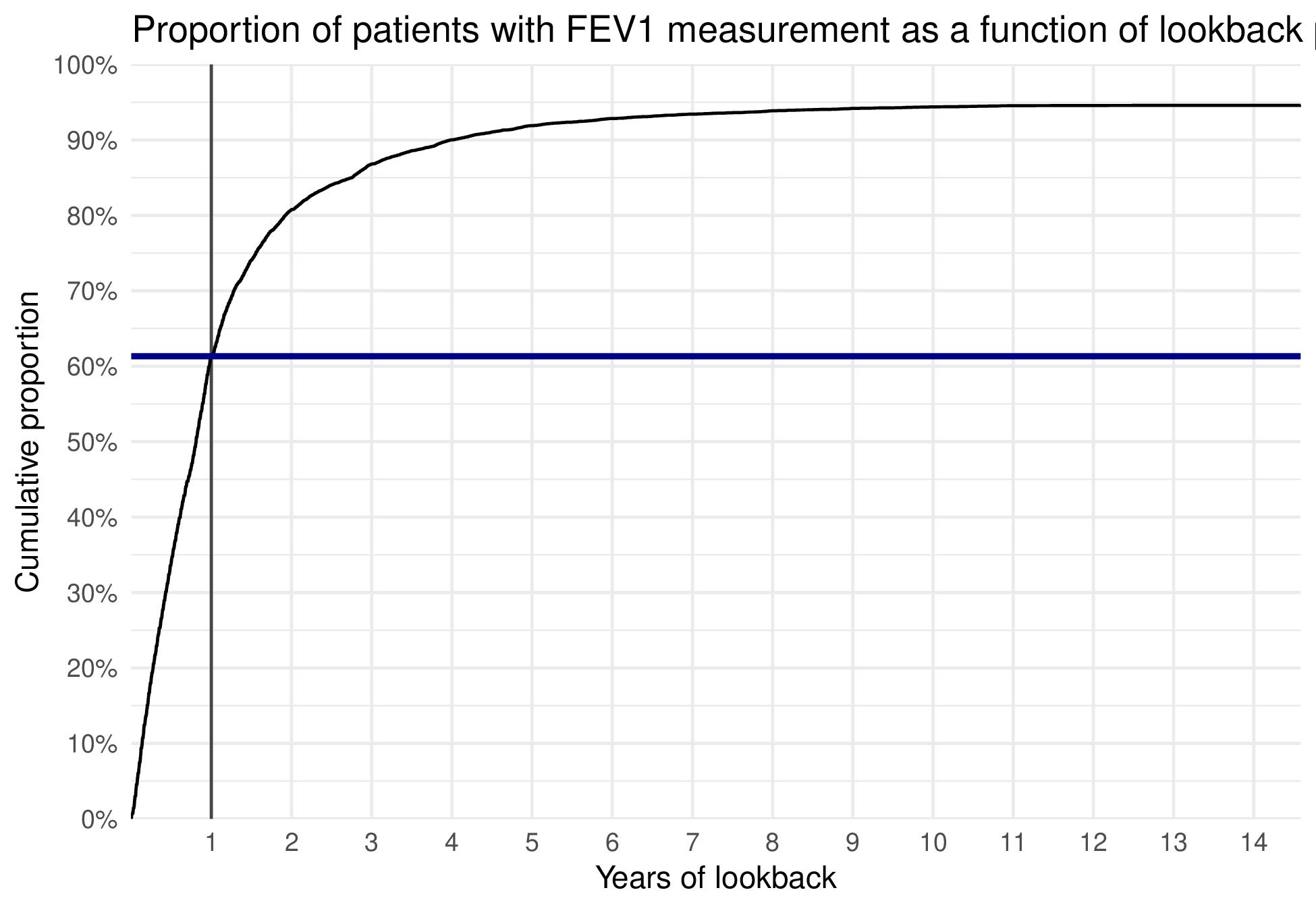


**Supplementary Figure S3 - The cumulative proportion of patients with a valid measurement of MRC as a function of the observation time. The blue line represents the proportion of patients with a valid measurement within one year prior to study entry (January 1st - December 31st 2014)**


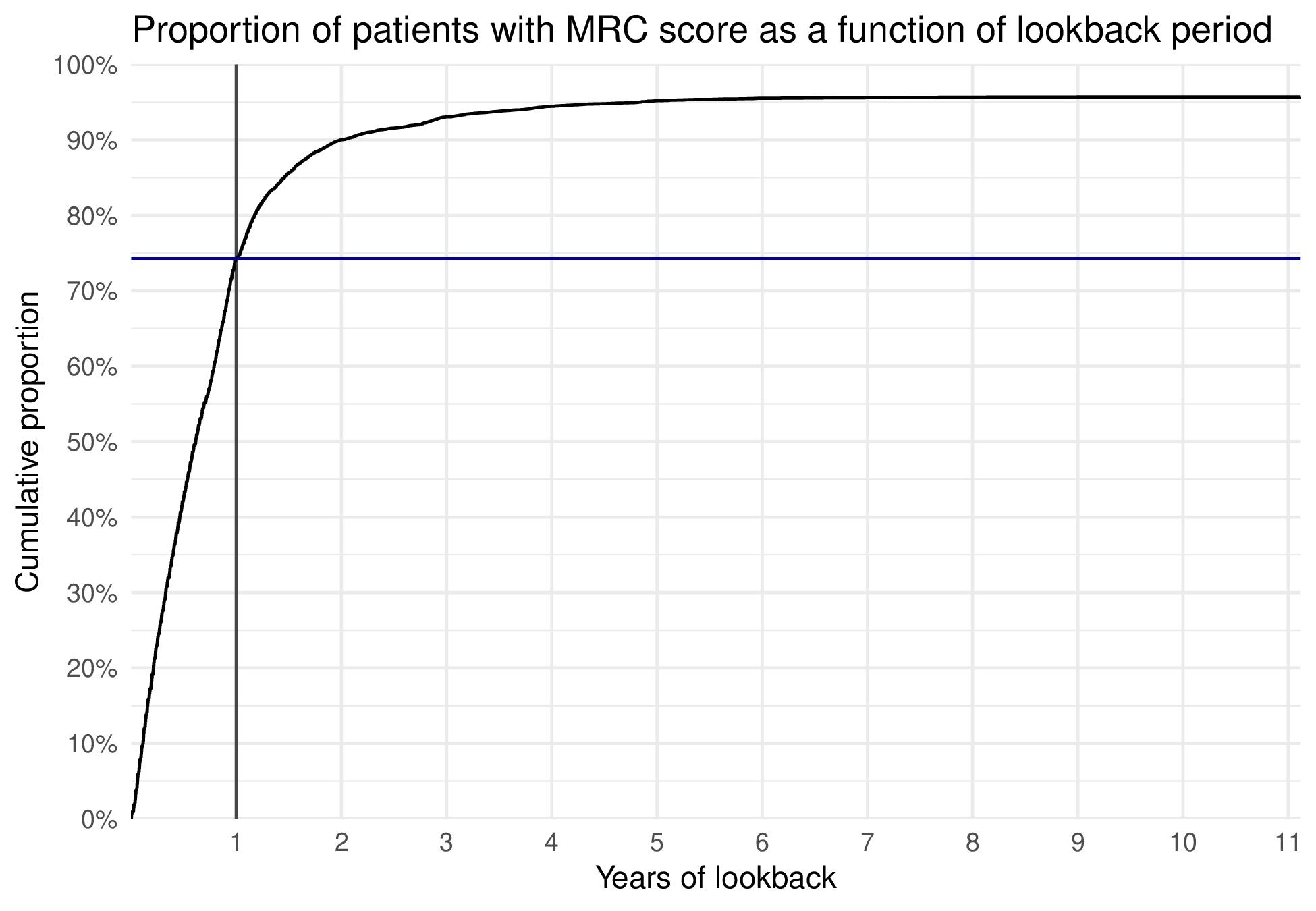


**Supplementary Table S7 - The (cumulative) proportion of patients with a valid measurement of FEV1 and/or MRC up to four years prior to index**

|  | Proportion of patients with a valid measure in the given year | | Cumulative proportion of patients with at least one measure in the given year or after | | |
| --- | --- | --- | --- | --- | --- |
|  | FEV1 | MRC | FEV1 | MRC | FEV1 or MRC |
| 0-1 years before index | 61.3% | 74.2% | 61.3% | 74.2% | 76.8% |
| 1-2 years before index | 58.7% | 67.5% | 80.8% | 90.0% | 91.9% |
| 2-3 years before index | 51.7% | 58.7% | 86.8% | 93.1% | 94.9% |
| 3-4 years before index | 47.1% | 52.9% | 90.0% | 94.5% | 96.3% |

**Supplementary Figure S4 - Correlation coefficient between FEV1 and MRC at baseline (0-1 year) and measures taken within two, three and four years. Pearson correlation was used for the absolute values of FEV1 and Spearman correlation for MRC score.**

##
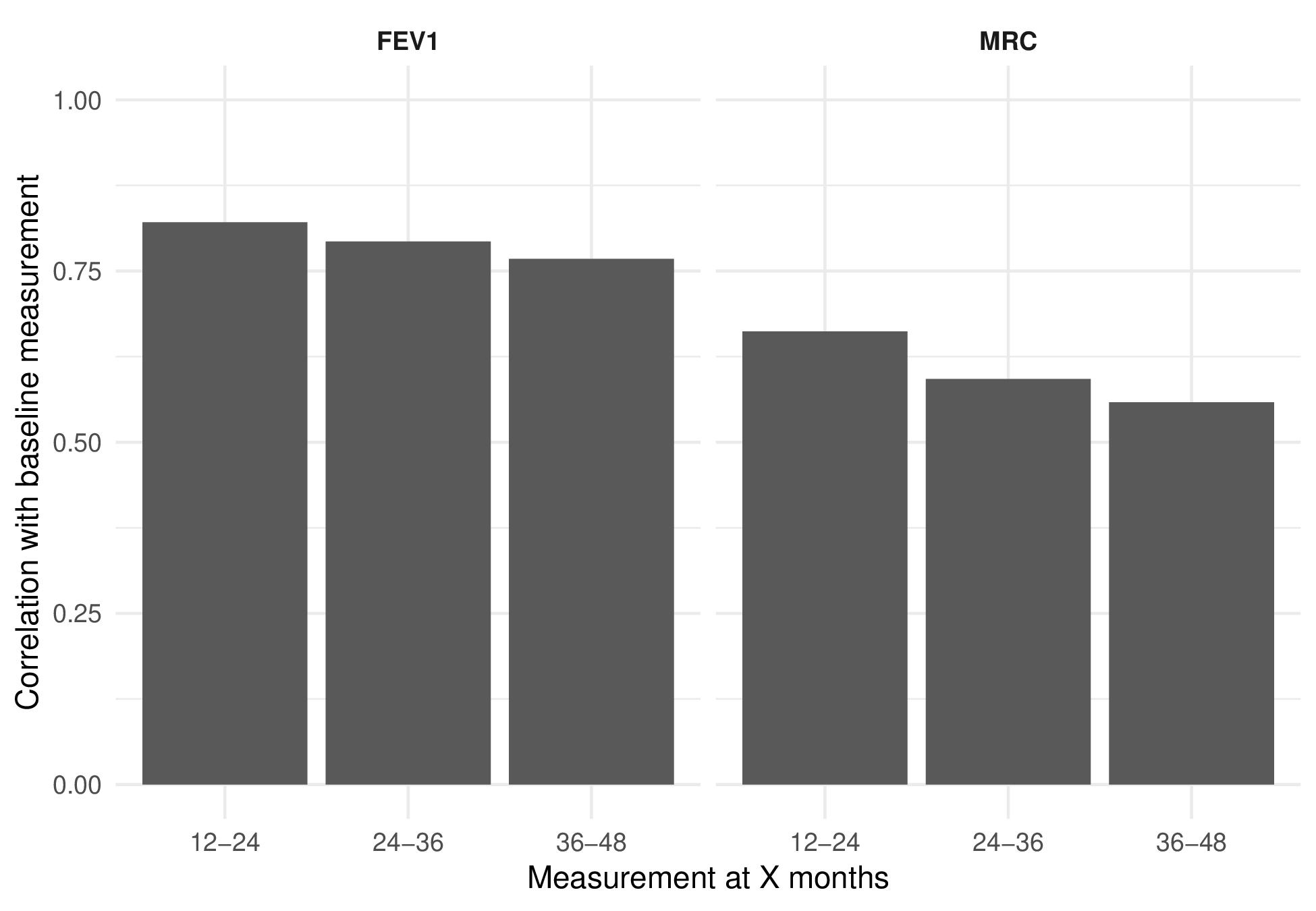


**Patterns of missingness**

Only 2,921 (14.9%) patients had a measurement each year for both FEV1 and MRC score (Supplementary Table 13). This might partially be due to the fact that patients that weren’t registered for a long time would not have had a measurement reaching back for 4 years.

**Supplementary Table S8 - The 20 most common patterns of missingness of FEV1 and MRC in the study cohort.**

| FEV1 available at  X months before index | | | |  | MRC score available at  X months before index | | | |  |  |
| --- | --- | --- | --- | --- | --- | --- | --- | --- | --- | --- |
| 36-48 | 24-36 | 12-24 | 0-12 |  | 36-48 | 24-36 | 12-24 | 0-12 | No. patients | % of patients |
| + | + | + | + |  | + | + | + | + | 2,921 | 14.9 |
| – | – | – | + |  | – | – | – | + | 1,323 | 6.8 |
| – | + | + | + |  | – | + | + | + | 1,086 | 5.5 |
| – | – | + | + |  | – | – | + | + | 861 | 4.4 |
| + | – | + | + |  | + | – | + | + | 859 | 4.4 |
| – | – | – | – |  | – | – | – | – | 730 | 3.7 |
| + | + | + | – |  | + | + | + | – | 675 | 3.4 |
| + | + | – | + |  | + | + | – | + | 603 | 3.1 |
| – | + | – | + |  | – | + | – | + | 482 | 2.5 |
| – | – | + | – |  | – | – | + | – | 443 | 2.3 |
| – | + | + | – |  | – | + | + | – | 401 | 2.0 |
| + | + | + | – |  | + | + | + | + | 365 | 1.9 |
| + | – | + | + |  | + | + | + | + | 345 | 1.8 |
| – | + | + | + |  | + | + | + | + | 329 | 1.7 |
| – | – | – | – |  | – | – | – | + | 297 | 1.5 |
| + | – | + | – |  | + | – | + | – | 297 | 1.5 |
| + | + | – | + |  | + | + | + | + | 247 | 1.3 |
| – | + | – | – |  | – | + | – | – | 213 | 1.1 |
| – | – | + | – |  | – | – | + | + | 207 | 1.1 |
| – | + | + | – |  | – | + | + | + | 191 | 1.0 |
|  |  |  |  |  |  |  |  |  |  |  |

**Distribution of non-missing values**

The distribution of FEV1 was at least approximately normal (Supplementary Figure 7). The cut-off at 100 was theoretical but wasn’t judged a problem as a problem as FEV1 was discretized later on. The distribution of MRC score was centered around 2-3, as expected (Supplementary Figure 8).

**Supplementary Figure S5 - Distribution of observed FEV1 values at baseline (theoretical normal distribution with the same mean and standard deviation overlaid in blue).**


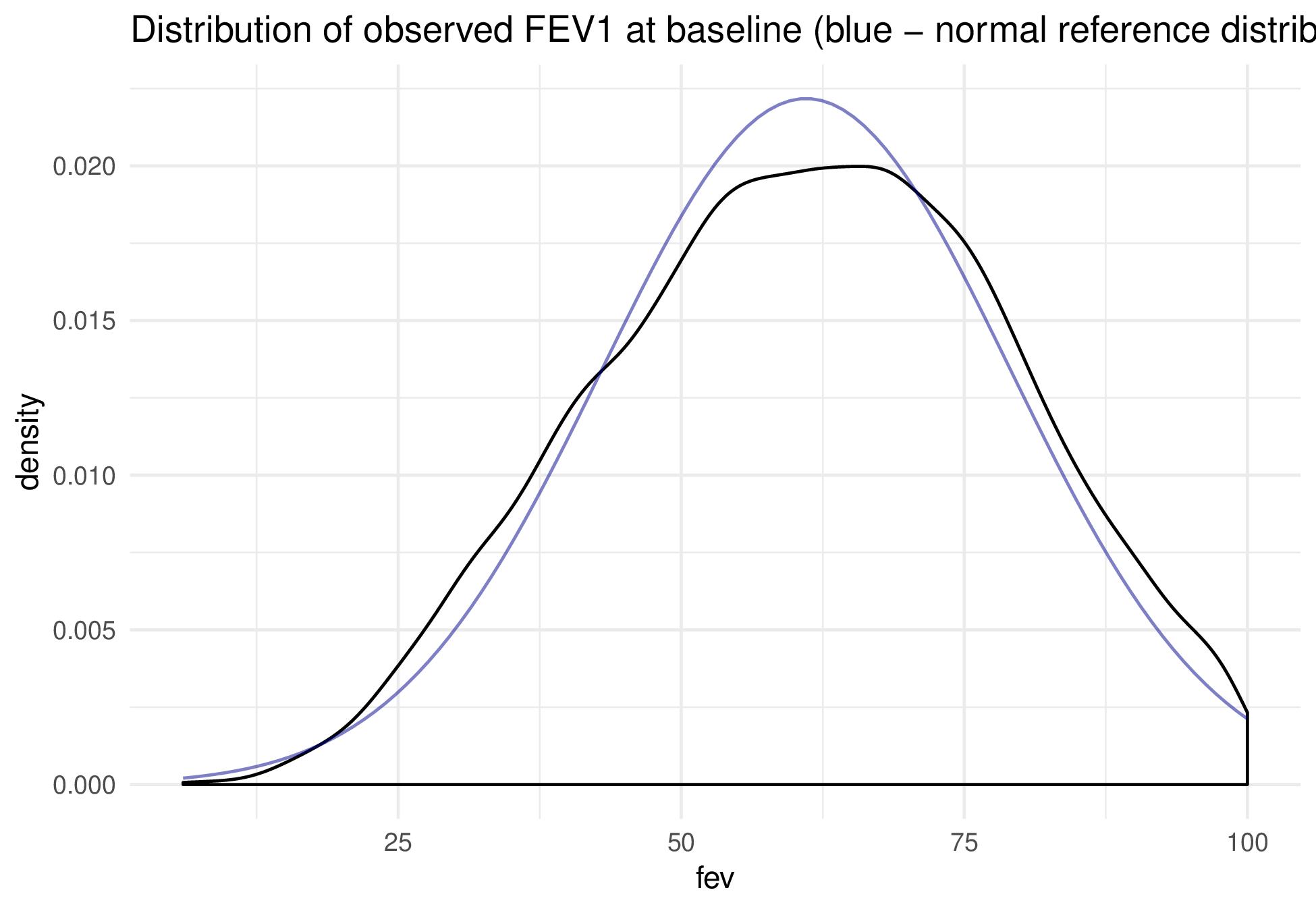


**Supplementary Figure S6 - Distribution of observed MRC values at baseline**


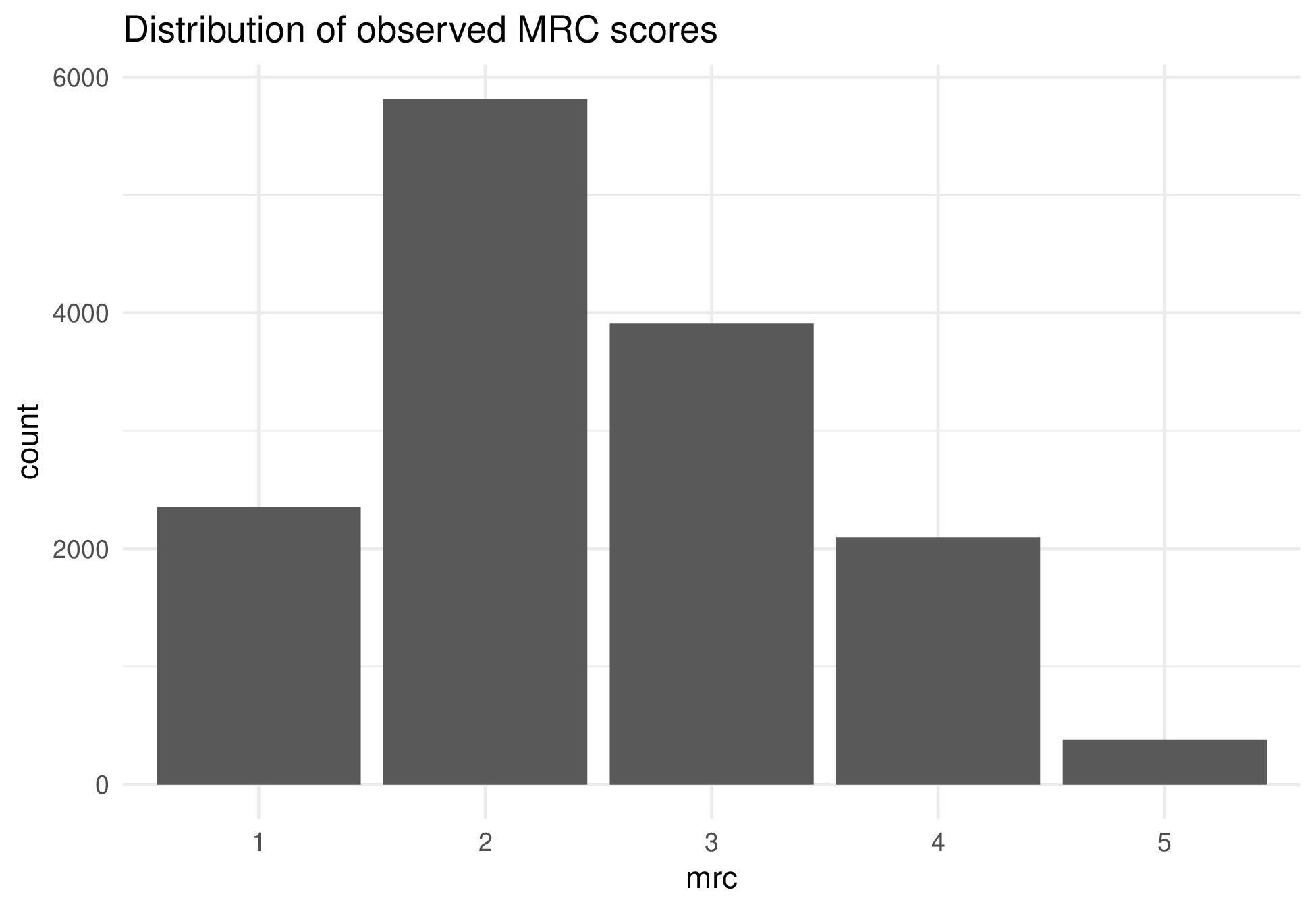


**Supplementary Table S9 - Associations of number of antibiotics, current and previous markers of COPD severity and covariates with missingness in FEV1 and MRC score**

|  |  |  |  |  |  |  |  | Odds of missingness in variable | | | | |
| --- | --- | --- | --- | --- | --- | --- | --- | --- | --- | --- | --- | --- |
|  | Whole dataset | | |  | Complete obs. | |  | FEV1 at baseline | |  | MRC at baseline | |
|  | No. with  measure | % with  measure | Mean value |  | No. with  measure | Mean value |  | OR | 95%-CI |  | OR | 95%-CI |
| **Main variables** |  |  |  |  |  |  |  |  |  |  |  |  |
| Number of antibiotics | 19,594 | 100 | 3.0 |  | 11,522 | 2.9 |  | 1.06 | 1.03-1.09 |  | 0.97 | 0.94-1.00 |
| FEV1 at baseline | 12,016 | 61 | 61.0 |  | 11,522 | 60.9 |  |  |  |  | 1.13 | 1.03-1.24 |
| MRC at baseline | 14,550 | 74 | 2.5 |  | 11,522 | 2.4 |  | 1.14 | 1.09-1.18 |  |  |  |
| AECOPD at baseline | 19,594 | 100 | 1.8 |  | 11,522 | 1.7 |  | 1.00 | 0.98-1.03 |  | 0.96 | 0.93-0.98 |
|  |  |  |  |  |  |  |  |  |  |  |  |  |
| **Past observations** |  |  |  |  |  |  |  |  |  |  |  |  |
| FEV1 - 36 months before | 9,243 | 47 | 60.7 |  | 6,022 | 60.2 |  | 1.09 | 1.04-1.14 |  | 1.05 | 1.00-1.11 |
| FEV1 - 24 months before | 10,126 | 52 | 61.0 |  | 6,719 | 60.5 |  | 1.08 | 1.04-1.13 |  | 1.05 | 1.00-1.10 |
| FEV1 - 12 months before | 11,496 | 59 | 61.0 |  | 7,480 | 60.6 |  | 1.07 | 1.03-1.12 |  | 1.03 | 0.98-1.08 |
| MRC - 36 months before | 10,378 | 53 | 2.4 |  | 6,485 | 2.4 |  | 1.07 | 1.03-1.12 |  | 1.04 | 0.99-1.09 |
| MRC - 24 months before | 11,513 | 59 | 2.4 |  | 7,230 | 2.4 |  | 1.08 | 1.04-1.12 |  | 1.02 | 0.98-1.07 |
| MRC - 23 months before | 13,228 | 68 | 2.4 |  | 8,044 | 2.4 |  | 1.07 | 1.03-1.11 |  | 1.00 | 0.96-1.04 |
|  |  |  |  |  |  |  |  |  |  |  |  |  |
| **Other variables** |  |  |  |  |  |  |  |  |  |  |  |  |
| Age | 19,594 | 100 | 70.6 |  | 11,522 | 70.6 |  | 1.02 | 0.99-1.05 |  | 0.90 | 0.87-0.93 |
| Sex | 19,594 | 100 | 46.4 |  | 11,522 | 44.5 |  | 1.20 | 1.13-1.27 |  | 1.13 | 1.06-1.20 |
| IMD | 19,594 | 100 | 3.2 |  | 11,522 | 3.2 |  | 1.06 | 1.03-1.08 |  | 1.06 | 1.03-1.08 |
| Asthma | 19,594 | 100 | 32.0 |  | 11,522 | 31.8 |  | 1.03 | 0.97-1.10 |  | 0.95 | 0.89-1.02 |
| CHD | 19,594 | 100 | 15.7 |  | 11,522 | 15.3 |  | 1.06 | 0.98-1.15 |  | 0.99 | 0.91-1.08 |
| CKD | 19,594 | 100 | 15.6 |  | 11,522 | 15.5 |  | 1.03 | 0.95-1.11 |  | 0.94 | 0.86-1.02 |
| Diabetes | 19,594 | 100 | 15.6 |  | 11,522 | 15.6 |  | 1.02 | 0.94-1.10 |  | 0.98 | 0.90-1.07 |
| Heart Failure | 19,594 | 100 | 5.6 |  | 11,522 | 5.4 |  | 1.11 | 0.98-1.25 |  | 1.09 | 0.95-1.25 |
| PAD | 19,594 | 100 | 6.0 |  | 11,522 | 5.9 |  | 1.08 | 0.96-1.22 |  | 0.97 | 0.85-1.11 |
| Stroke | 19,594 | 100 | 8.4 |  | 11,522 | 7.9 |  | 1.21 | 1.10-1.35 |  | 1.13 | 1.01-1.26 |
| Obesity | 19,594 | 100 | 28.5 |  | 11,522 | 29.4 |  | 0.89 | 0.84-0.95 |  | 0.87 | 0.81-0.94 |
| Smoking status | 19,594 | 100 | 39.1 |  | 11,522 | 37.4 |  | 1.17 | 1.11-1.24 |  | 1.33 | 1.25-1.42 |
|  |  |  |  |  |  |  |  |  |  |  |  |  |

**Supplementary Table S10 - Associations of current and previous markers of COPD severity and covariates with the number of antibiotics received in 2015**

|  | Whole dataset | | |  | Complete obs. | |  | Number of antibiotics | |
| --- | --- | --- | --- | --- | --- | --- | --- | --- | --- |
|  | No. with  measure | % with  measure | Mean value |  | No. with  measure | Mean value |  | RR | 95%-CI |
| **Main variables** |  |  |  |  |  |  |  |  |  |
| FEV1 at baseline | 12,016 | 61 | 61.0 |  | 11,522 | 60.9 |  | 0.81 | 0.80-0.81 |
| MRC at baseline | 14,550 | 74 | 2.5 |  | 11,522 | 2.4 |  | 1.39 | 1.38-1.40 |
| AECOPD at baseline | 19,594 | 100 | 1.8 |  | 11,522 | 1.7 |  | 1.36 | 1.35-1.37 |
|  |  |  |  |  |  |  |  |  |  |
| **Past observations** |  |  |  |  |  |  |  |  |  |
| FEV1 - 36 months before | 9,243 | 47 | 60.7 |  | 6,022 | 60.2 |  | 0.83 | 0.82-0.84 |
| FEV1 - 24 months before | 10,126 | 52 | 61.0 |  | 6,719 | 60.5 |  | 0.82 | 0.81-0.83 |
| FEV1 - 12 months before | 11,496 | 59 | 61.0 |  | 7,480 | 60.6 |  | 0.80 | 0.80-0.81 |
| MRC - 36 months before | 10,378 | 53 | 2.4 |  | 6,485 | 2.4 |  | 1.32 | 1.31-1.33 |
| MRC - 24 months before | 11,513 | 59 | 2.4 |  | 7,230 | 2.4 |  | 1.35 | 1.34-1.36 |
| MRC - 23 months before | 13,228 | 68 | 2.4 |  | 8,044 | 2.4 |  | 1.36 | 1.34-1.37 |
|  |  |  |  |  |  |  |  |  |  |
| **Other variables** |  |  |  |  |  |  |  |  |  |
| Age | 19,594 | 100 | 70.6 |  | 11,522 | 70.6 |  | 1.04 | 1.03-1.05 |
| Sex | 19,594 | 100 | 46.4 |  | 11,522 | 44.5 |  | 1.22 | 1.20-1.24 |
| IMD | 19,594 | 100 | 3.2 |  | 11,522 | 3.2 |  | 1.03 | 1.02-1.04 |
| Asthma | 19,594 | 100 | 32.0 |  | 11,522 | 31.8 |  | 1.29 | 1.27-1.31 |
| CHD | 19,594 | 100 | 15.7 |  | 11,522 | 15.3 |  | 1.19 | 1.16-1.21 |
| CKD | 19,594 | 100 | 15.6 |  | 11,522 | 15.5 |  | 1.12 | 1.10-1.15 |
| Diabetes | 19,594 | 100 | 15.6 |  | 11,522 | 15.6 |  | 1.18 | 1.16-1.21 |
| Heart Failure | 19,594 | 100 | 5.6 |  | 11,522 | 5.4 |  | 1.35 | 1.31-1.40 |
| PAD | 19,594 | 100 | 6.0 |  | 11,522 | 5.9 |  | 1.08 | 1.05-1.12 |
| Stroke | 19,594 | 100 | 8.4 |  | 11,522 | 7.9 |  | 1.14 | 1.11-1.17 |
| Obesity | 19,954 | 100 | 28.5 |  | 11,522 | 29.4 |  | 1.15 | 1.13-1.17 |
| Smoking status | 19,594 | 100 | 39.1 |  | 11,522 | 37.4 |  | 0.89 | 0.87-0.90 |
|  |  |  |  |  |  |  |  |  |  |

**Supplementary Figure S7 - Convergence of the mean and standard deviation of 20 chains of FEV1 and MRC at 0 (baseline), 12, 24, and 36 months after 40 iterations with multiple imputation using chained equations**

**
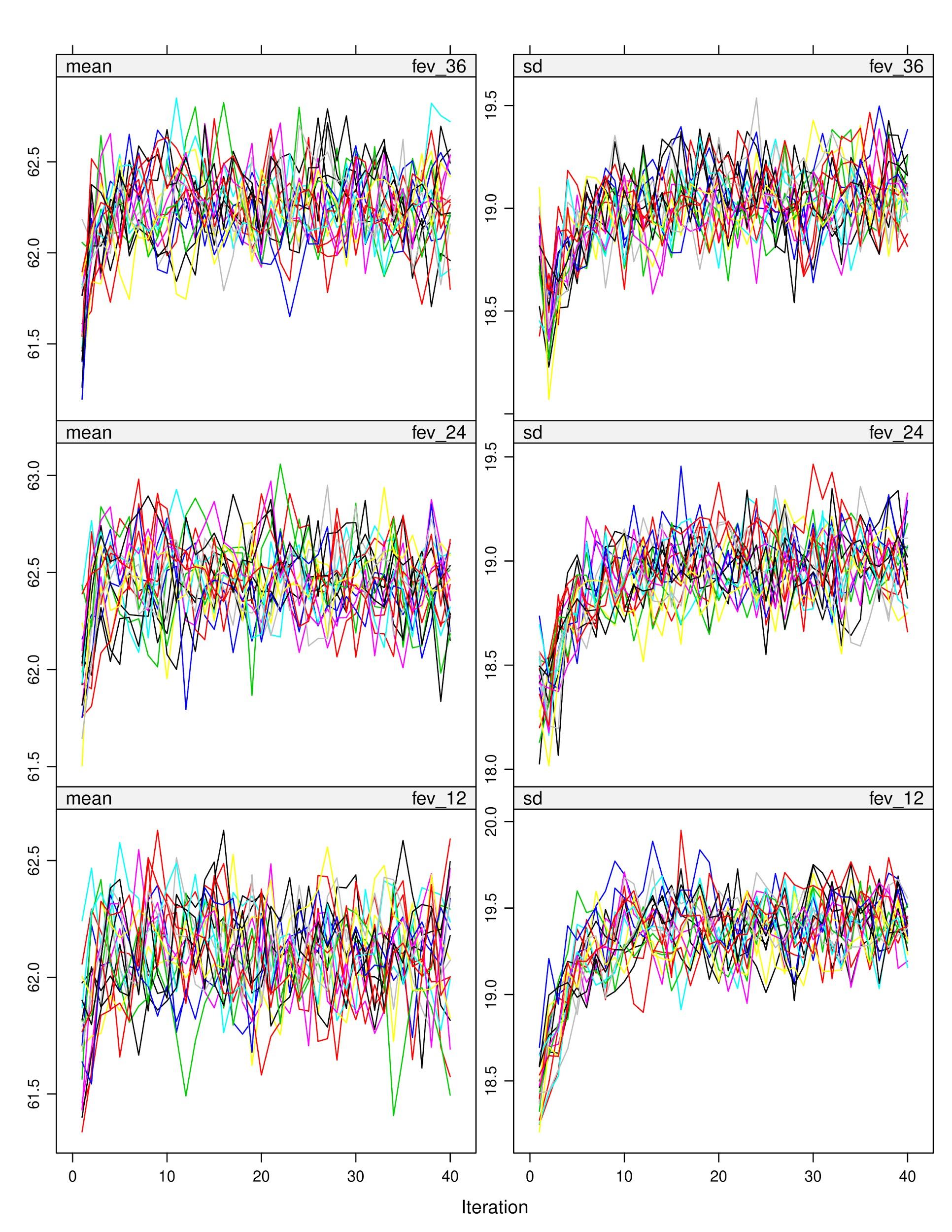
**

**
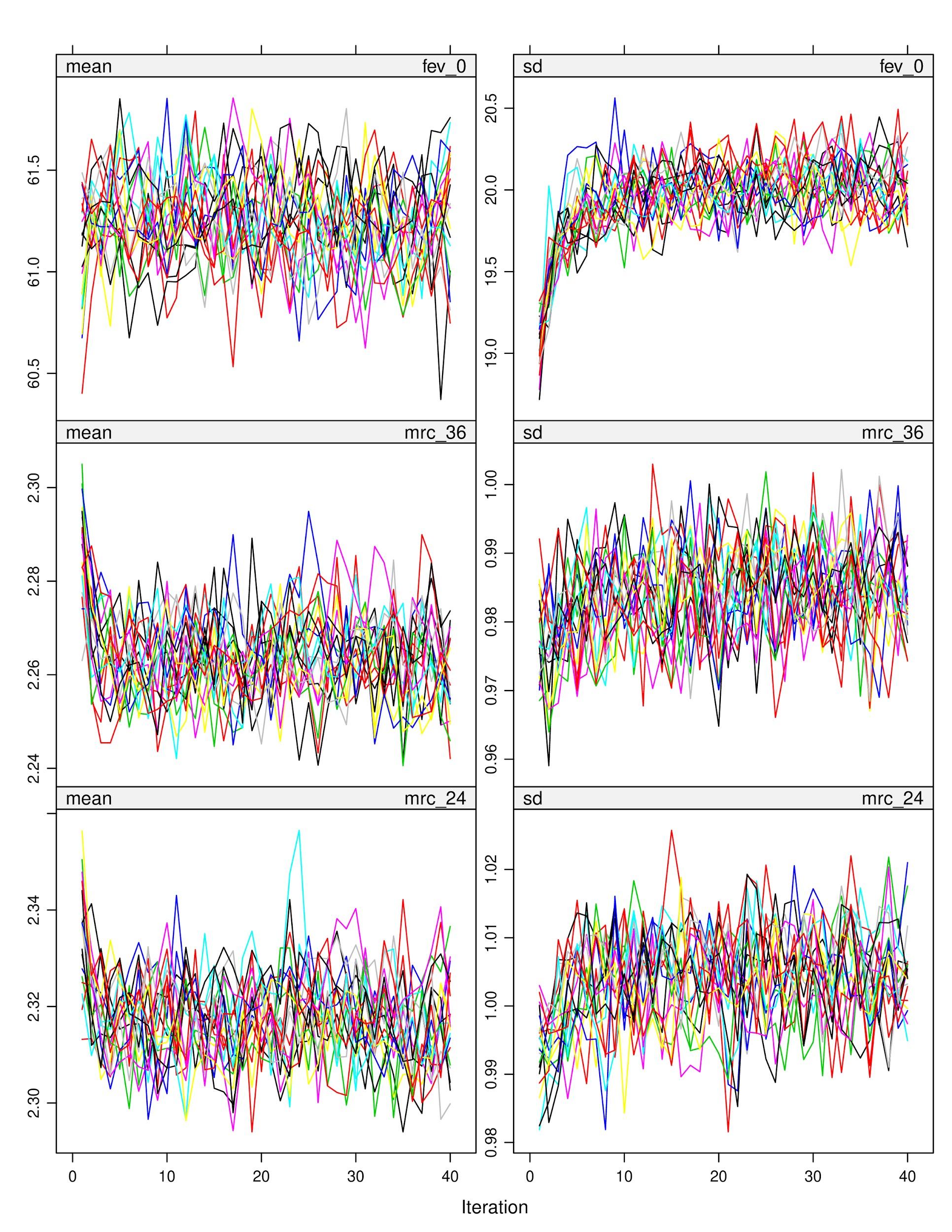
**

**
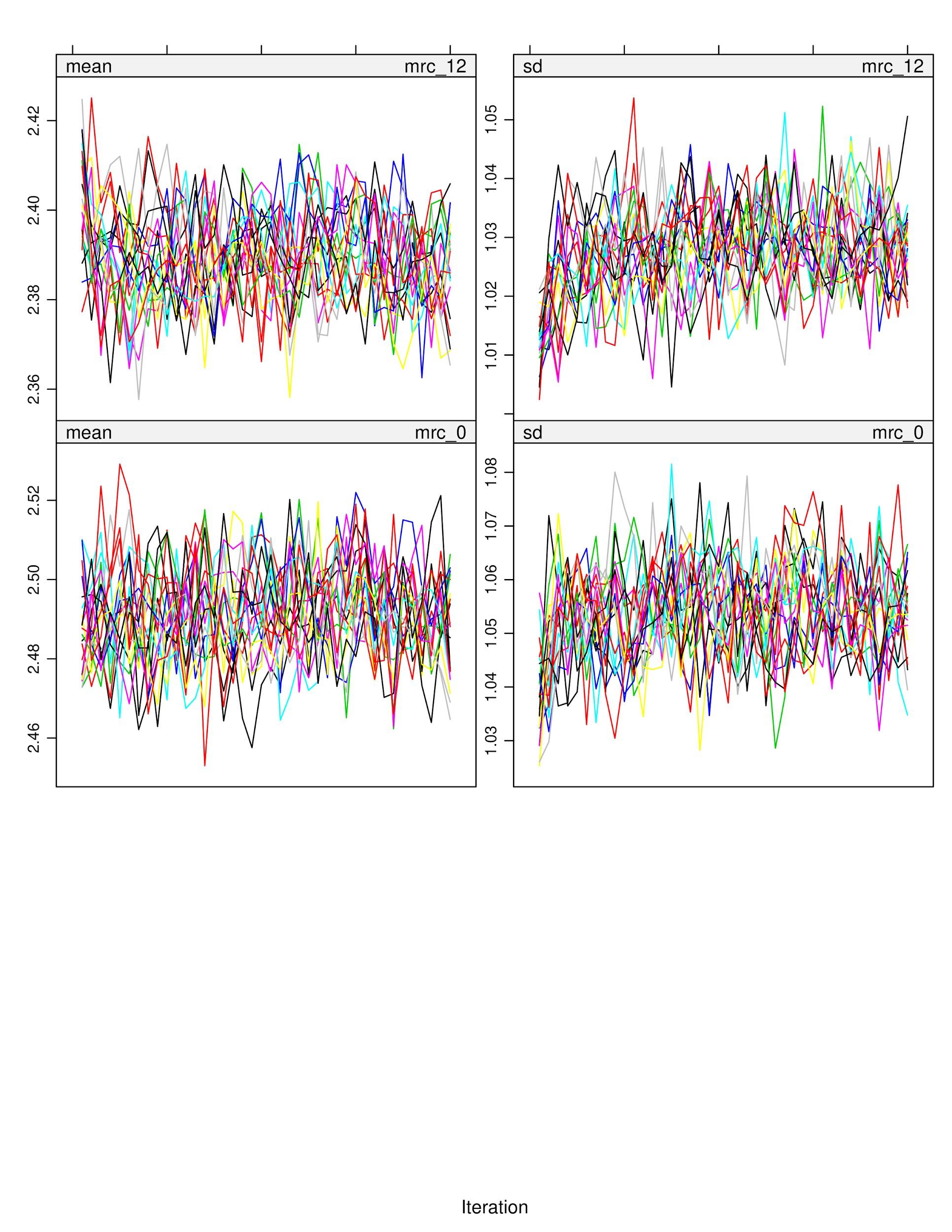
**

**Supplementary Table S11 - Average number of patients in each combination of COPD severity and number of AECOPD during follow-up after multiple imputation**

|  | Number of AECOPD during follow-up | | | | |
| --- | --- | --- | --- | --- | --- |
|  |  | Primary care | | | Hospital |
|  | 0 | 1 | 2 | ≥3 | ≥1 |
|  | n | n | n | n | n |
| **FEV1 according to GOLD criteria*** | | |  |  |  |
| GOLD 1 | 2,293 | 503 | 167 | 129 | 166 |
| GOLD 2 | 7,216 | 1,686 | 622 | 540 | 733 |
| GOLD 3 | 2,636 | 709 | 288 | 256 | 585 |
| GOLD 4 | 556 | 151 | 60 | 66 | 233 |
|  |  |  |  |  |  |
| **MRC breathlessness score*** | |  |  |  |  |
| MRC 1 | 2,484 | 456 | 118 | 84 | 93 |
| MRC 2 | 5,320 | 1,210 | 440 | 328 | 447 |
| MRC 3 | 3,140 | 834 | 352 | 335 | 537 |
| MRC 4 | 1,485 | 468 | 194 | 208 | 510 |
| MRC 5 | 272 | 81 | 34 | 36 | 129 |
|  |  |  |  |  |  |
| **Number of AECOPD during baseline** | | |  |  |  |
| 0 | 9,420 | 1,690 | 452 | 212 | 683 |
| 1 in primary care | 1,931 | 706 | 267 | 195 | 275 |
| 2 in primary care | 527 | 170 | 99 | 95 | 485 |
| ≥3 in primary care | 556 | 291 | 149 | 156 | 118 |
| ≥1 in hospital | 267 | 192 | 170 | 333 | 155 |
|  |  |  |  |  |  |

* Missing values imputed and mean number of patients in each cell rounded to the nearest natural number. Columns might not add up exactly due to small rounding errors.

**Additional results**

**Supplementary Table S12 - Multivariate analysis of the association between the baseline characteristics, FEV1 and AECOPD and rate of antibiotic prescribing in the following 12 months**

|  |  | FEV1 | AECOPD during baseline |
| --- | --- | --- | --- |
| Patient characteristics | Unadj. RR  (95%-CI) | Adj. RR  (95%-CI) | Adj. RR  (95%-CI) |
| **Total** |  |  |  |
| **Age** |  |  |  |
| 35-50 | 0.80 (0.71-0.90) | 0.84 (0.71-0.99) | 0.82 (0.70-0.96) |
| 50-60 | 0.87 (0.81-0.93) | 0.88 (0.80-0.96) | 0.85 (0.78-0.93) |
| 60-70* | 1 | 1 | 1 |
| 70-80 | 1.02 (0.97-1.07) | 1.03 (0.97-1.10) | 1.02 (0.95-1.08) |
| >80 | 1.02 (0.96-1.08) | 1.04 (0.96-1.12) | 0.97 (0.90-1.08) |
| **Female** | 1.23 (1.19-1.28) | 1.29 (1.20-1.38) | 1.23 (1.15-1.31) |
| **Index of Multiple deprivation** |  |  |  |
| Q1 (least deprived) | 0.93 (0.87-1.00) | 0.95 (0.88-1.01) | 0.96 (0.90-1.02) |
| Q2 | 0.94 (0.88-1.01) | 0.96 (0.90-1.02) | 0.95 (0.90-1.01) |
| Q3* | 1 | 1 | 1 |
| Q4 | 1.05 (0.99-1.12) | 1.06 (1.00-1.13) | 1.04 (0.98-1.10) |
| Q5 (most deprived) | 1.06 (0.99-1.13) | 1.07 (1.00-1.14) | 1.04 (0.98-1.11) |
| **FEV1 according to GOLD criteria** |  |  |  |
| GOLD 1 | 0.86 (0.81-0.93) | 0.84 (0.78-0.91) |  |
| GOLD 2* | 1 | 1 |  |
| GOLD 3 | 1.38 (1.31-1.46) | 1.41 (1.32-1.50) |  |
| GOLD 4 | 1.94 (1.74-2.17) | 2.05 (1.85-2.27) |  |
|  |  |  |  |
| **MRC breathlessness score** |  |  |  |
| MRC 1 | 0.70 (0.65-0.75) |  |  |
| MRC 2* | 1 |  |  |
| MRC 3 | 1.45 (1.37-1.53) |  |  |
| MRC 4 | 1.99 (1.87-2.12) |  |  |
| MRC 5 | 2.61 (2.29-2.96) |  |  |
|  |  |  |  |
| **Frequency of AECOPD at baseline** |  |  |  |
| 0* | 1 |  | 1 |
| 1 in primary care | 1.56 (1.48-1.64) |  | 1.52 (1.45-1.60) |
| 2 in primary care | 2.20 (2.05-2.36) |  | 2.16 (2.01-2.32) |
| ≥3 in primary care | 3.33 (3.09-3.59) |  | 3.22 (2.99-3.47) |
| ≥1 in hospital | 3.21 (3.00-3.44) |  | 3.05 (2.86-3.27) |
|  |  |  |  |
| **Asthma** | 1.30 (1.25-1.36) | 1.26 (1.21-1.31) | 1.19 (1.14-1.24) |
| **CHD** | 1.18 (1.12-1.25) | 1.16 (1.10-1.23) | 1.11 (1.05-1.16) |
| **CKD** | 1.15 (1.09-1.22) | 1.06 (0.98-1.15) | 1.06 (1.01-1.12) |
| **Diabetes** | 1.18 (1.12-1.24) | 1.10 (1.04-1.16) | 1.10 (1.05-1.16) |
| **Heart Failure** | 1.37 (1.26-1.49) | 1.24 (1.14-1.34) | 1.15 (1.06-1.24) |
| **PAD** | 1.09 (1.01-1.18) | 1.06 (0.98-1.15) | 1.01 (0.93-1.09) |
| **Stroke** | 1.14 (1.07-1.23) | 1.09 (1.02-1.17) | 1.09 (1.02-1.17) |
| **Obesity** | 1.14 (1.10-1.19) | 1.13 (1.08-1.18) | 1.07 (1.03-1.12) |
| **Smoking** | 0.87 (0.84-0.91) | 0.91 (0.87-0.95) | 0.91 (0.88-0.95) |
| **Flu vaccination** | 1.27 (1.21-1.34) | 1.26 (1.20-1.33) | 1.23 (1.18-1.29) |
|  |  |  |  |

* Reference category

**Supplementary Table S13 - Complete case analysis of the rate of antibiotic prescribing according to the number of AECOPD during follow-up for FEV1 and MRC score**

|  | Number of AECOPD during follow-up | | | | |
| --- | --- | --- | --- | --- | --- |
|  |  | Primary care | | | Hospital |
|  | 0 | 1 | 2 | ≥3 | ≥1 |
|  | Rate (95%-CI) | Rate (95%-CI) | Rate (95%-CI) | Rate (95%-CI) | Rate (95%-CI) |
| **FEV1 according to GOLD criteria** | |  |  |  |  |
| GOLD 1 | 1.20 (1.10-1.32) | 2.55 (2.22-2.94) | 4.70 (3.71-5.95) | 6.05 (4.72-7.74) | 5.14 (4.02-6.57) |
| GOLD 2 | 1.36 (1.28-1.45) | 3.00 (2.76-3.27) | 4.53 (4.02-5.11) | 6.45 (5.70-7.28) | 5.21 (4.62-5.87) |
| GOLD 3 | 1.90 (1.76-2.05) | 3.44 (3.07-3.85) | 5.38 (4.58-6.33) | 7.20 (6.10-8.51) | 5.78 (5.12-6.52) |
| GOLD 4 | 3.12 (2.71-3.59) | 4.50 (3.54-5.72) | 4.83 (3.24-7.19) | 9.33 (6.65-13.10) | 6.74 (5.54-8.19) |
|  |  |  |  |  |  |
| **MRC breathlessness score** |  |  |  |  |  |
| MRC 1 | 1.01 (0.93-1.09) | 2.58 (2.26-2.95) | 3.91 (3.06-4.99) | 6.25 (4.79-8.16) | 4.65 (3.53-6.12) |
| MRC 2 | 1.37 (1.29-1.46) | 2.95 (2.71-3.22) | 4.16 (3.67-4.71) | 6.66 (5.79-7.65) | 4.79 (4.23-5.44) |
| MRC 3 | 1.88 (1.75-2.01) | 3.50 (3.17-3.85) | 5.25 (4.59-6.01) | 7.19 (6.29-8.23) | 6.37 (5.69-7.13) |
| MRC 4 | 2.72 (2.50-2.95) | 4.35 (3.85-4.92) | 6.20 (5.17-7.43) | 8.61 (7.29-10.18) | 6.93 (6.17-7.78) |
| MRC 5 | 3.74 (3.16-4.42) | 4.90 (3.70-6.50) | 7.16 (4.62-11.08) | 8.58 (5.79-12.72) | 9.13 (7.36-11.33) |
|  |  |  |  |  |  |

**Supplementary Figure S8 - Relationship between disease severity (assessed by FEV1), rate of antibiotic prescribing according to the number of AECOPD. Tile sizes are scaled to reflect the proportion of antibiotics that were prescribed to patients in each group.**


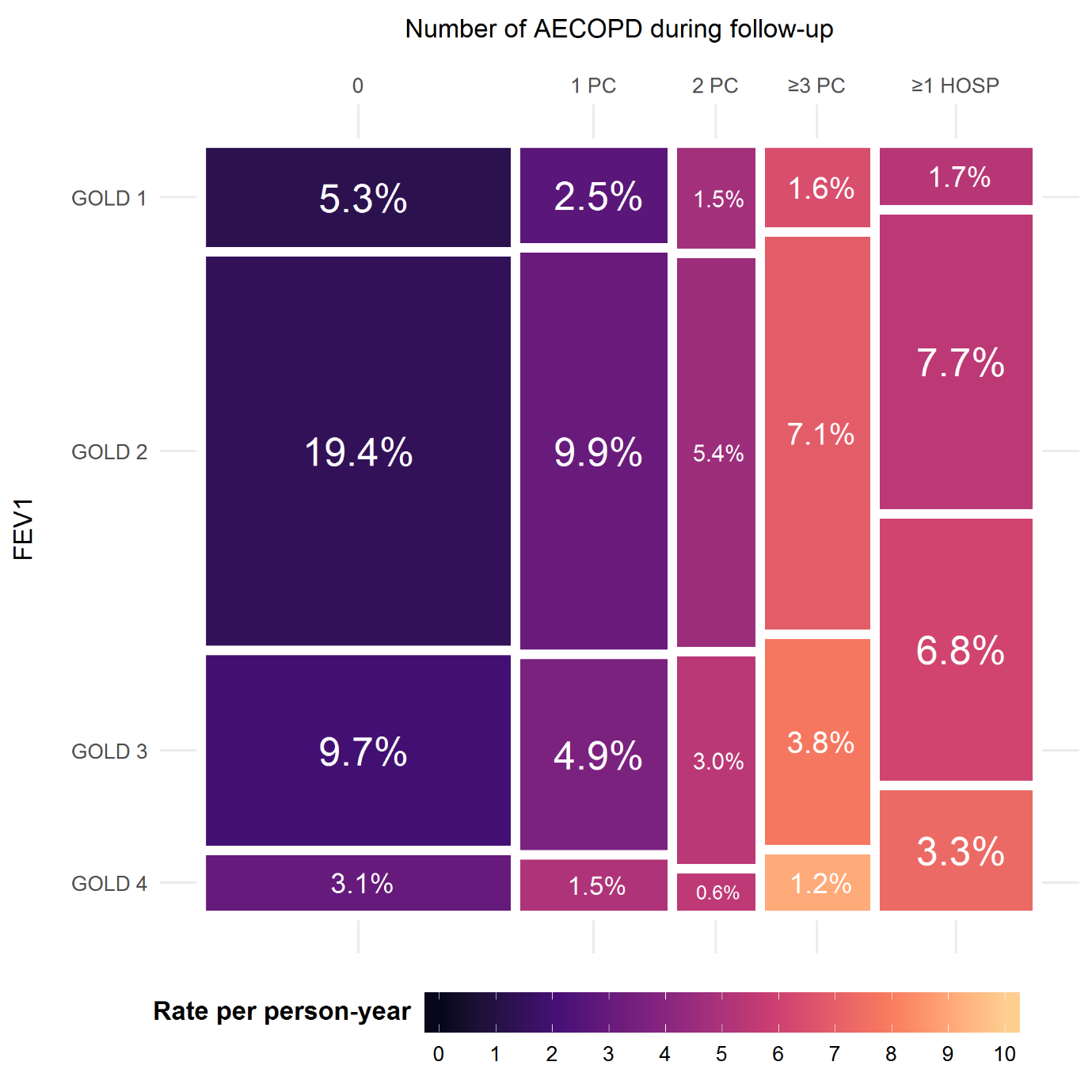


PC: AECOPD managed in primary care, HOSP: AECOPD requiring hospitalisation

**Supplementary Figure S9 - Relationship between disease severity (assessed by the number of AECOPD during baseline) and the rate of antibiotic prescribing according to the number of AECOPD. Tile sizes are scaled to reflect the proportion of antibiotics that were prescribed to patients in each group.**

**
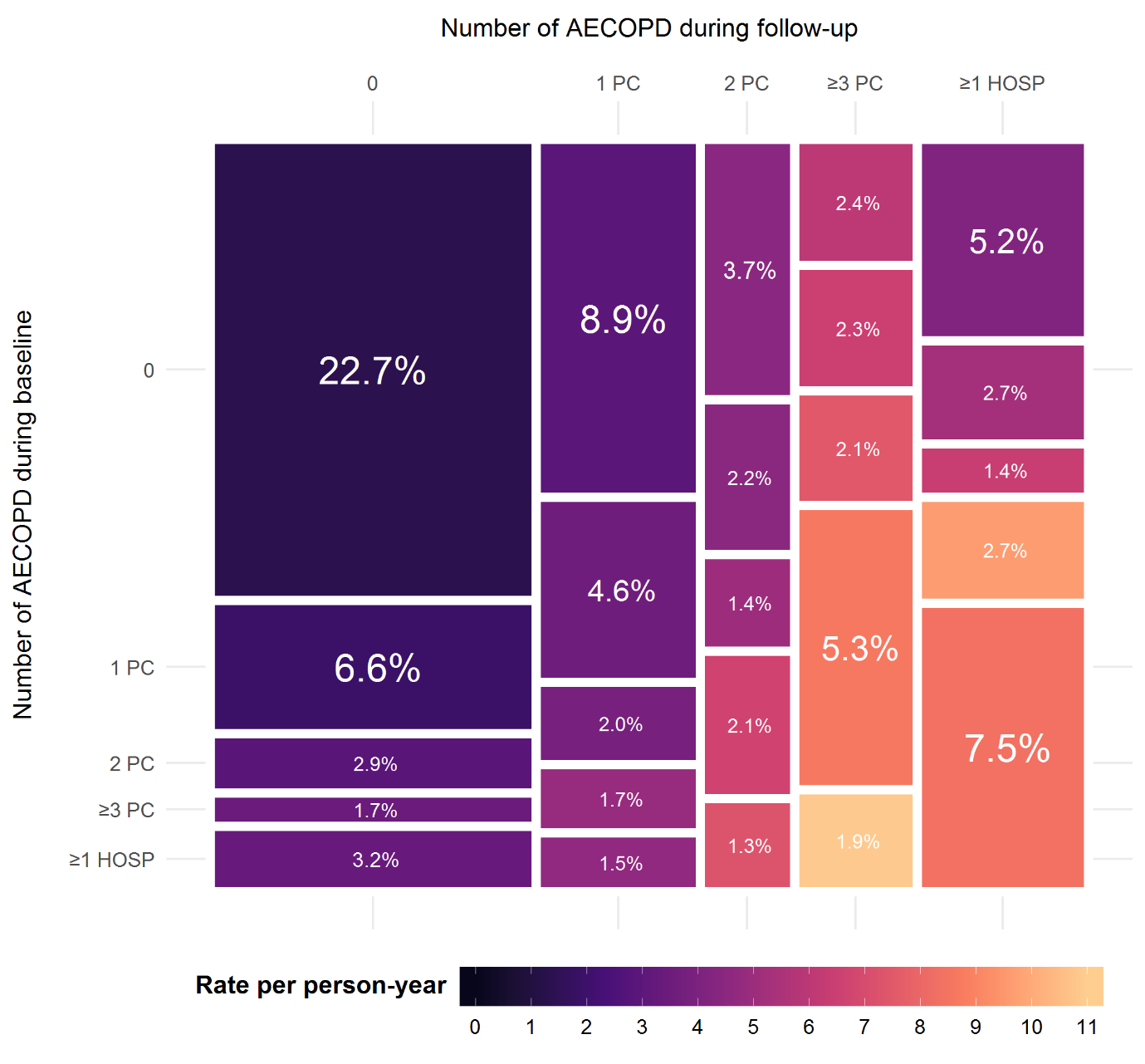
**

PC: AECOPD managed in primary care, HOSP: AECOPD requiring hospitalisation

**Sensitivity analysis**

**Supplementary Table S14 - Complete case analysis of the rate of antibiotic prescribing according to the number of AECOPD during follow-up for 2013**

|  | Number of AECOPD during follow-up | | | | |
| --- | --- | --- | --- | --- | --- |
|  |  | Primary care | | | Hospital |
|  | 0 | 1 | 2 | ≥3 | ≥1 |
|  | Rate (95%-CI) | Rate (95%-CI) | Rate (95%-CI) | Rate (95%-CI) | Rate (95%-CI) |
| **FEV1 according to GOLD criteria** | |  |  |  |  |
| GOLD 1 | 1.28 (1.20-1.37) | 2.53 (2.29-2.80) | 4.30 (3.68-5.02) | 6.37 (5.44-7.45) | 4.38 (3.60-5.33) |
| GOLD 2 | 1.37 (1.31-1.43) | 2.81 (2.65-2.98) | 4.20 (3.88-4.55) | 7.05 (6.49-7.66) | 4.57 (4.19-4.99) |
| GOLD 3 | 1.65 (1.56-1.74) | 3.04 (2.82-3.28) | 4.12 (3.69-4.59) | 7.41 (6.68-8.21) | 5.55 (5.07-6.07) |
| GOLD 4 | 2.39 (2.15-2.67) | 4.50 (3.86-5.24) | 5.38 (4.24-6.83) | 8.81 (7.24-10.71) | 7.12 (6.21-8.16) |
| **MRC breathlessness score** |  |  |  |  |  |
| MRC 1 | 1.02 (0.96-1.08) | 2.28 (2.08-2.51) | 3.90 (3.39-4.49) | 6.38 (5.43-7.49) | 3.83 (3.11-4.72) |
| MRC 2 | 1.27 (1.21-1.33) | 2.73 (2.57-2.90) | 4.02 (3.69-4.37) | 6.49 (5.93-7.10) | 4.29 (3.87-4.74) |
| MRC 3 | 1.80 (1.71-1.89) | 3.27 (3.05-3.50) | 4.52 (4.11-4.97) | 7.33 (6.69-8.03) | 5.14 (4.72-5.60) |
| MRC 4 | 2.38 (2.24-2.53) | 3.88 (3.57-4.22) | 4.99 (4.44-5.61) | 8.43 (7.60-9.35) | 6.53 (6.00-7.10) |
| MRC 5 | 3.21 (2.82-3.64) | 4.83 (3.98-5.87) | 6.19 (4.69-8.18) | 10.42 (8.10-13.42) | 7.57 (6.54-8.77) |
| **Number of AECOPD during baseline** | |  |  |  |  |
| 0 | 1.22 (1.18-1.27) | 2.58 (2.45-2.71) | 3.92 (3.62-4.24) | 5.86 (5.30-6.47) | 3.44 (3.20-3.70) |
| 1 in primary care | 1.85 (1.76-1.95) | 3.18 (2.98-3.39) | 4.23 (3.87-4.63) | 6.59 (5.97-7.27) | 4.99 (4.49-5.55) |
| 2 in primary care | 2.09 (1.93-2.26) | 3.43 (3.14-3.75) | 4.50 (4.00-5.05) | 7.17 (6.41-8.01) | 5.74 (5.00-6.58) |
| ≥3 in primary care | 2.97 (2.70-3.28) | 4.19 (3.79-4.63) | 5.12 (4.59-5.71) | 8.39 (7.76-9.08) | 7.91 (7.03-8.90) |
| ≥1 in hospital | 3.21 (2.96-3.48) | 4.29 (3.80-4.84) | 5.28 (4.49-6.21) | 9.91 (8.58-11.46) | 7.30 (6.77-7.87) |
|  |  |  |  |  |  |

**Supplementary Table S15 - Complete case analysis of the rate of antibiotic prescribing according to the number of AECOPD during follow-up for 2014**

|  | Number of AECOPD during follow-up | | | | |
| --- | --- | --- | --- | --- | --- |
|  |  | Primary care | | | Hospital |
|  | 0 | 1 | 2 | ≥3 | ≥1 |
|  | Rate (95%-CI) | Rate (95%-CI) | Rate (95%-CI) | Rate (95%-CI) | Rate (95%-CI) |
| **FEV1 according to GOLD criteria** | |  |  |  |  |
| GOLD 1 | 1.17 (1.09-1.25) | 2.55 (2.28-2.85) | 3.88 (3.25-4.62) | 6.94 (5.78-8.35) | 4.77 (3.96-5.76) |
| GOLD 2 | 1.39 (1.33-1.46) | 2.84 (2.67-3.04) | 4.29 (3.92-4.70) | 6.57 (5.98-7.21) | 5.25 (4.78-5.77) |
| GOLD 3 | 1.85 (1.74-1.96) | 3.08 (2.82-3.37) | 4.72 (4.19-5.32) | 7.53 (6.72-8.43) | 5.67 (5.14-6.25) |
| GOLD 4 | 2.61 (2.32-2.94) | 4.29 (3.56-5.18) | 5.82 (4.44-7.64) | 7.30 (5.69-9.37) | 7.47 (6.44-8.67) |
| **MRC breathlessness score** |  |  |  |  |  |
| MRC 1 | 1.03 (0.97-1.10) | 2.23 (2.00-2.48) | 3.36 (2.85-3.97) | 6.24 (5.13-7.59) | 3.49 (2.76-4.41) |
| MRC 2 | 1.35 (1.29-1.42) | 2.71 (2.53-2.90) | 4.17 (3.78-4.60) | 6.87 (6.23-7.58) | 4.78 (4.30-5.32) |
| MRC 3 | 1.80 (1.70-1.90) | 3.28 (3.04-3.54) | 4.68 (4.21-5.20) | 7.14 (6.45-7.90) | 5.81 (5.31-6.36) |
| MRC 4 | 2.35 (2.20-2.51) | 3.91 (3.54-4.31) | 5.46 (4.78-6.23) | 8.09 (7.13-9.17) | 7.33 (6.68-8.05) |
| MRC 5 | 3.35 (2.93-3.83) | 4.79 (3.76-6.08) | 7.84 (5.70-10.77) | 8.74 (6.50-11.74) | 7.56 (6.31-9.06) |
| **Number of AECOPD during baseline** | |  |  |  |  |
| 0 | 1.27 (1.21-1.32) | 2.62 (2.47-2.77) | 4.05 (3.71-4.42) | 5.73 (5.11-6.42) | 4.12 (3.80-4.46) |
| 1 in primary care | 1.86 (1.75-1.97) | 3.02 (2.80-3.25) | 4.65 (4.20-5.14) | 6.58 (5.87-7.37) | 5.61 (5.01-6.28) |
| 2 in primary care | 2.58 (2.37-2.81) | 3.38 (3.05-3.76) | 4.40 (3.84-5.04) | 7.21 (6.35-8.20) | 6.79 (5.83-7.90) |
| ≥3 in primary care | 3.11 (2.77-3.48) | 4.61 (4.10-5.18) | 5.90 (5.20-6.71) | 8.72 (7.96-9.55) | 9.69 (8.53-11.01) |
| ≥1 in hospital | 3.04 (2.78-3.32) | 4.53 (3.96-5.19) | 6.01 (4.93-7.32) | 8.88 (7.53-10.46) | 7.31 (6.71-7.96) |
|  |  |  |  |  |  |
